# Age-related changes in eye lens biomechanics, morphology, refractive index and transparency

**DOI:** 10.18632/aging.102584

**Published:** 2019-12-16

**Authors:** Catherine Cheng, Justin Parreno, Roberta B. Nowak, Sondip K. Biswas, Kehao Wang, Masato Hoshino, Kentaro Uesugi, Naoto Yagi, Juliet A. Moncaster, Woo-Kuen Lo, Barbara Pierscionek, Velia M. Fowler

**Affiliations:** 1 School of Optometry, Indiana University, Bloomington, IN 47405, USA; 2 Department of Molecular Medicine, The Scripps Research Institute, La Jolla, CA 92037, USA; 3 Department of Biological Sciences, University of Delaware, Newark, DE 19716, USA; 4 Department of Neurobiology, Morehouse School of Medicine, Atlanta, GA 30303, USA; 5 School of Science and Technology, Nottingham Trent University, Nottingham, United Kingdom; 6 Japan Synchrotron Radiation Research Institute (Spring-8), Sayo-cho, Sayo-gun, Hyogo, Japan; 7 Department of Radiology, Boston University School of Medicine, Boston, MA 02118, USA

**Keywords:** fiber cell, strain, epithelial cell, cataract, stiffness

## Abstract

Life-long eye lens function requires an appropriate gradient refractive index, biomechanical integrity and transparency. We conducted an extensive study of wild-type mouse lenses 1-30 months of age to define common age-related changes. Biomechanical testing and morphometrics revealed an increase in lens volume and stiffness with age. Lens capsule thickness and peripheral fiber cell widths increased between 2 to 4 months of age but not further, and thus, cannot account for significant age-dependent increases in lens stiffness after 4 months. In lenses from mice older than 12 months, we routinely observed cataracts due to changes in cell structure, with anterior cataracts due to incomplete suture closure and a cortical ring cataract corresponding to a zone of compaction in cortical lens fiber cells. Refractive index measurements showed a rapid growth in peak refractive index between 1 to 6 months of age, and the area of highest refractive index is correlated with increases in lens nucleus size with age. These data provide a comprehensive overview of age-related changes in murine lenses, including lens size, stiffness, nuclear fraction, refractive index, transparency, capsule thickness and cell structure. Our results suggest similarities between murine and primate lenses and provide a baseline for future lens aging studies.

## INTRODUCTION

The eye lens is required for fine focusing of light onto the retina to form a clear image, and the function of the lens is intimately tied to its shape, biomechanical properties, transparency and refractive index. It has long been known that age-related changes in these lens properties lead to two major lens pathologies, cataracts and presbyopia [1]. Cataracts, defined as any lens opacity, are the leading cause of blindness in the world [2], and almost all mammalian and avian species develop age-related cataracts [3–15]. Changes in lens transparency occur in several locations, cortical, subcapsular and nuclear, and there are many hypothesized causes for opacities, including UV light exposure, reactive oxygen species, nutrition and genetic variations [16–18]. Though previous studies have demonstrated that wild-type mouse lenses develop age-related cataracts [10, 12–15, 19, 20], the age at which defects appear and the mechanisms for opacities in different locations in lenses from aged mice has not been thoroughly studied. Presbyopia is caused by a reduction in the lens’ ability to change shape during focusing (accommodation), and, by extension, the need for reading glasses [21–23]. Studies have linked age-related increases in lens stiffness to presbyopia [21–26], and increased lens stiffness with age has been reported in humans [21, 26–34] and animal models [34–36], including mice [37–41].

The lens is composed of two cell types, a monolayer of epithelial cells covering the anterior hemisphere and a bulk mass of differentiated lens fiber cells (Supplementary Figure 1). A basement membrane, called the capsule, encapsulates the lens [42]. Life-long lens growth occurs through the proliferation, elongation and differentiation of equatorial epithelial cells into new fiber cells that are added in concentric layers surrounding previous generations of fibers [43–45]. Hexagonal packing of lens fibers is established by cell reorganization and shape changes in equatorial epithelial cells [46, 47]. High refractive index in the lens is established by the high concentration and short range order of crystallin proteins [48, 49]. Previous studies, including our work, suggest that cytoskeletal structures are important to maintain lens mechanical integrity [38, 40, 50, 51]. The lens is a unique organ where all the cells are retained since the formation of the tissue during embryonic development, and there is little or no protein turnover in cells at the center of the lens [42]. This presents a rare opportunity to study cellular aging by allowing comparison of cells made in the embryo vs. cells added during in adulthood vs. cells formed in old age.

Little is known about the morphological, mechanical, refractive and cellular changes that occur with advanced age in the lens. Mice offer an opportunity to investigate changes in lens morphometrics, stiffness, transparency and refractive properties with age in a relatively shortened period of time. We carried out a comprehensive study of the properties of mouse lenses from young adult mice (1–2 months old) to very old mice (24–30 months old), measuring size, Gradient Refractive Index (GRIN) and stiffness. We also determined that anterior subcapsular and cortical ring cataracts appear in wild-type mouse lenses around 12 months of age. Confocal and electron microscopy revealed morphological changes in lens fiber cells that are correlated with these changes in lens transparency, suggesting a cellular basis for these age-related cataracts. In all, we demonstrate that age-related changes in mouse lenses mimic some aspects of aging in human lenses.

## RESULTS

### Mouse lenses have increased size, nuclear fraction, stiffness and resilience with age

**Figure 1:**
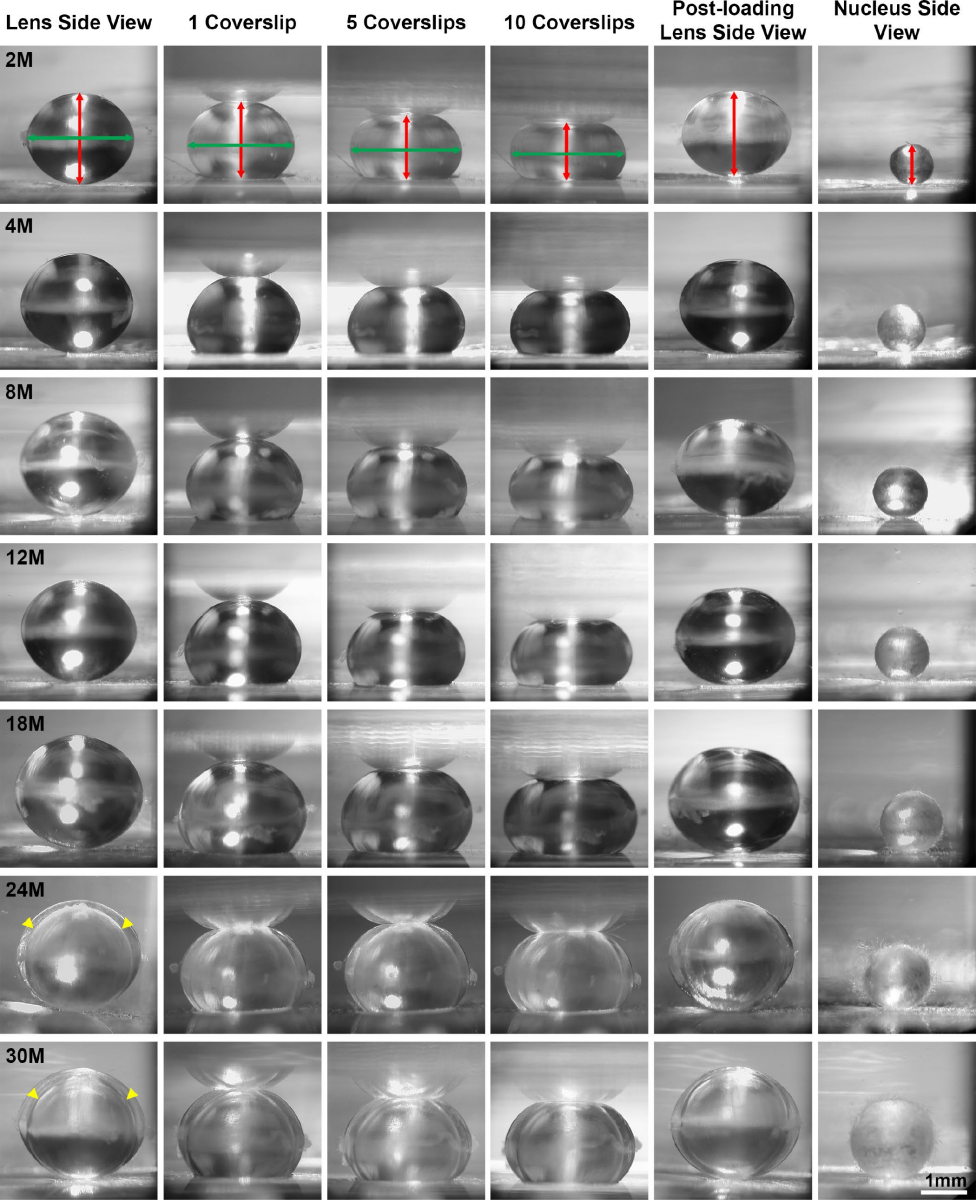
**Side view pictures of mouse lenses between 2-30 months of age pre-compression, during coverslip compression (1, 5 and 10 coverslips) and post-compression, and the isolated lens nucleus.** With age, the application of the same load compressed the older lenses less than young lenses. There is an overall increase in lens size and nucleus size with age. The axial diameter (red double-headed arrows) and the equatorial diameter (green double-headed arrows) for each lens were measured to calculate lens volume, lens aspect ratio, axial compressive strain, equatorial expansion strain, resilience and nuclear volume. In very old lenses (24-30 months), there is an area of optical discontinuity in the lens cortex (yellow arrowheads). Scale bar, 1mm.

**Figure 2:**
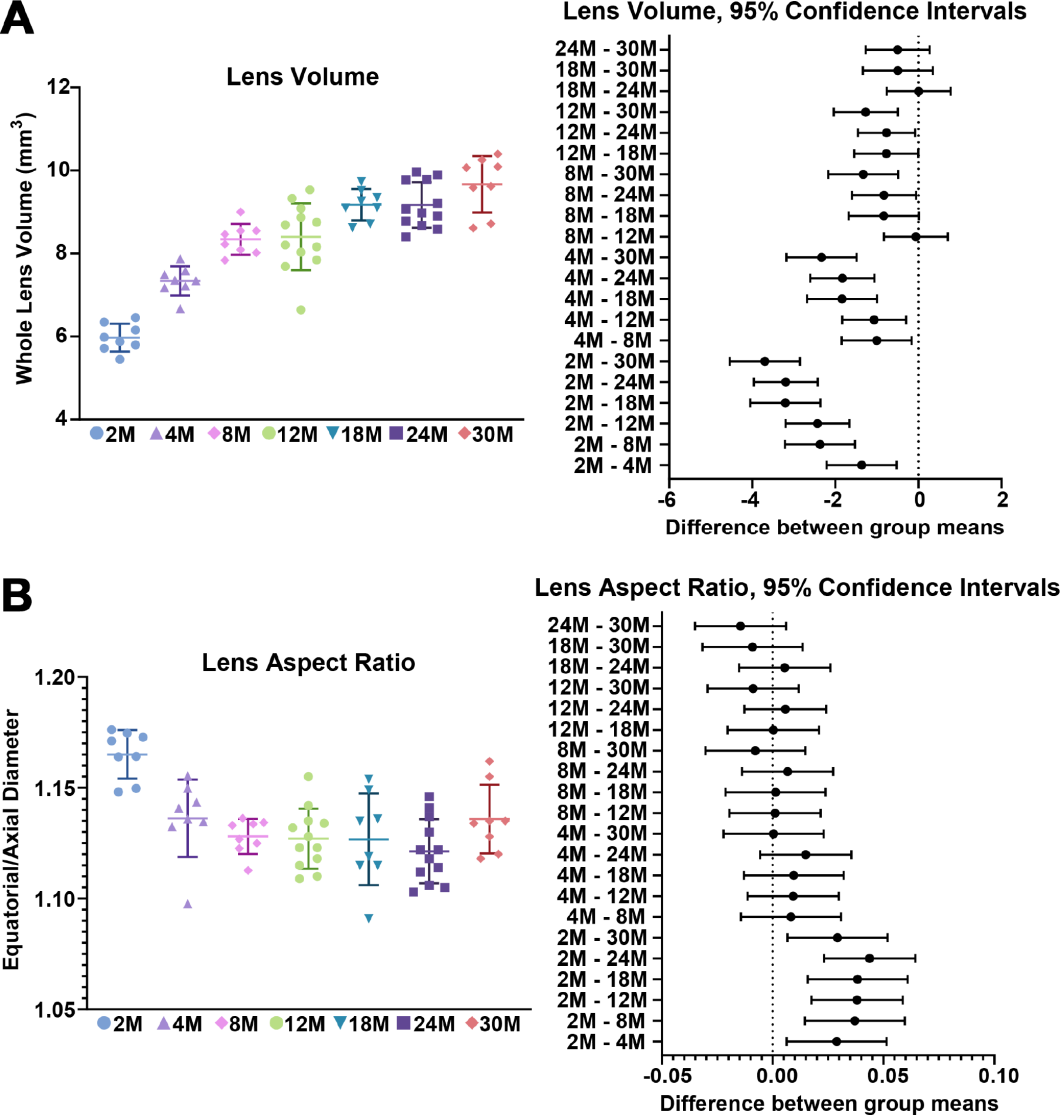
**Lens volume and aspect ratio for mouse lenses between 2–30 months of age.** Lines on the plots reflect mean ± SD of n = at least 8 lenses per age. The graph next to the data plots shows the 95% confidence interval. Any comparisons not crossing the dotted line are statistically significant (p < 0.05). (**A**) Lens volume (mm^3^) from mice between 2–30 months of age. The volume increases steadily between 2-8 months of age and more slowly after 8 months. (**B**) The lens aspect ratio (axial/equatorial diameter) drops slightly between 2 to 4 months of age and then remains unchanged with age. Mouse lenses become slightly more spherical between 2 and 4 months.

**Figure 3:**
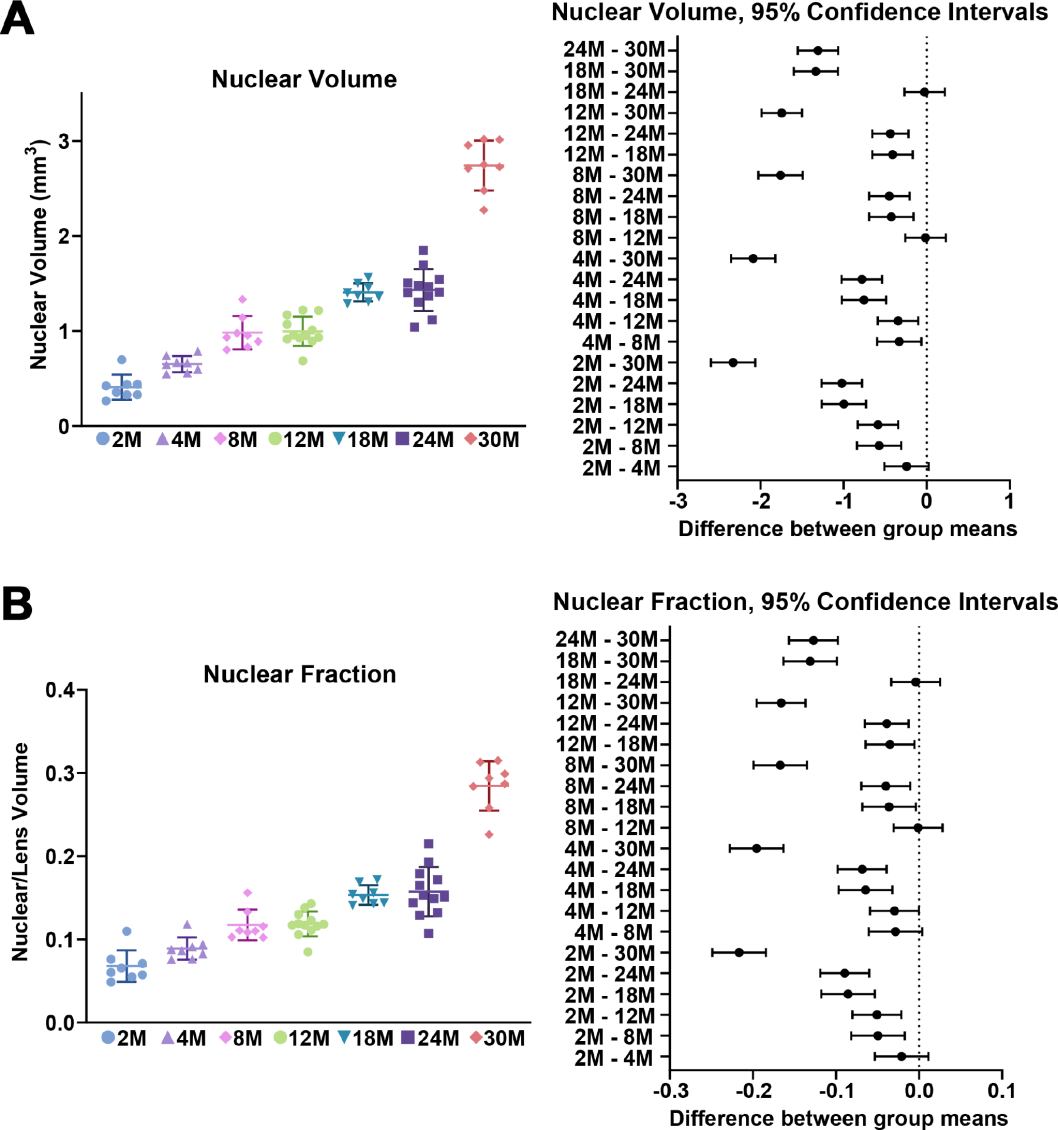
**Nuclear volume and fraction for mouse lenses between 2–30 months of age.** Lines on the plots reflect mean ± SD of n = at least 8 lenses per age. The graph next to the data plots shows the 95% confidence interval. Any comparisons not crossing the dotted line are statistically significant (p < 0.05). (**A**) The volume (mm^3^) of the lens nucleus steadily increases with age. (**B**) Since nuclear volume increases more than lens volume with age, the nuclear fraction (nuclear/lens volume) increases with age.

We first examined the morphometrics of mouse lenses between 2–30 months of age (Figure 1). We measured the axial (Figure 1, red double-headed arrows) and equatorial diameters (Figure 2, green double-headed) arrows for each lens to calculate lens volume, lens aspect ratio, nuclear volume and biomechanical properties. We observed that lenses from very old mice (24–30 months) have an optical discontinuity in the lens cortex (Figure 1, yellow arrowheads). For morphometric measurements, we used dot plots with lines to show the average and standard deviation, and also included a graph for each parameter of the Tukey 95% confidence interval from the statistical analysis, to assess the significance of the multiple comparisons between the age groups. The horizontal lines representing each comparison are statistically significant if they do not cross the vertical dotted line (95% confidence), which is similar in concept to a *p*-value less than 0.05. We found that lenses steadily increased in volume between 2 to 8 months of age, with growth slowing down between 8 to 18 months of age (Figures 1 and 2A). Mouse lenses do not increase significantly in volume after 18 months of age. The equatorial diameter of lenses does not increase significantly after 8 months of age while axial diameter increases slightly until 18 months of age (data not shown). Mouse lenses are nearly spherical in shape, becoming slightly more spherical between 2 and 4 months of age (Figure 2B). After 4 months, the lens aspect ratio does not change significantly with age. Lens fiber cells at the center, or nucleus, of the lens become compacted [21, 52], and in mouse lenses, the nucleus is a hard and spherical structure that can be isolated by removing the softer cells of the lens cortex [38, 41]. In contrast to the constant lens volume after 18 months of age, the size of the nucleus continues to increase with age up to 30 months of age (Figures 1 and 3). While the rate of increase is slow (no change from 8 to 12 months, or from 18 to 24 months), the size of the nucleus increases significantly between 24 to 30 months of age. These data suggest that while overall lens growth slows down, the remodelling and compaction of the nucleus continues in the mouse lens with age.

**Figure 4:**
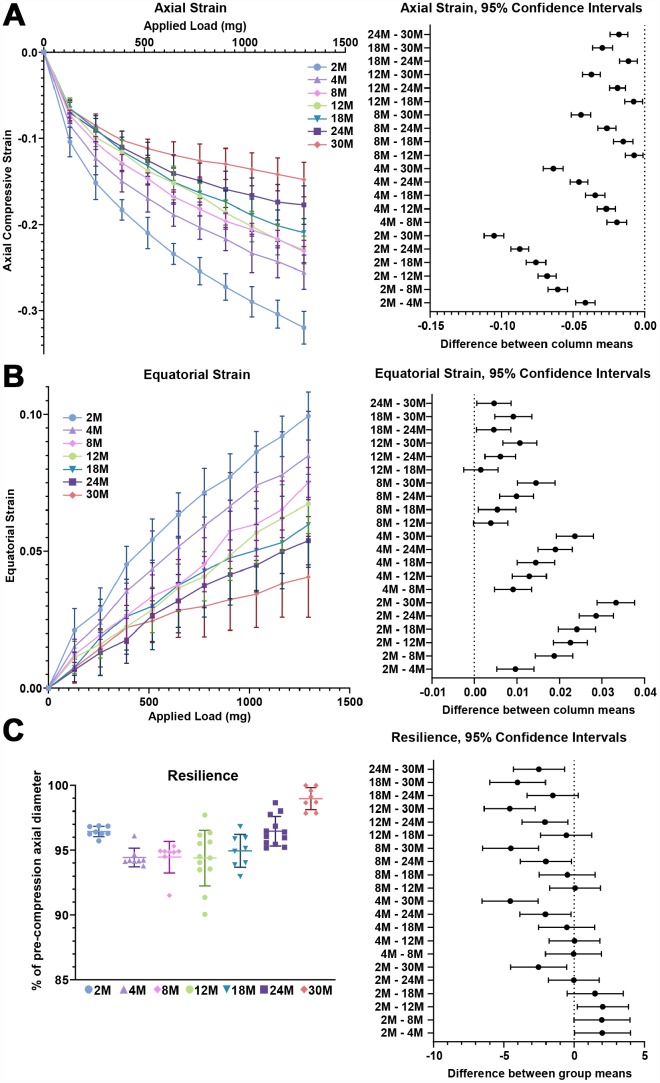
**Lens stiffness and resilience for mouse lenses between 2–30 months of age.** Plots reflect mean ± SD of n = at least 8 lenses per age. The graph next to the data plots shows the 95% confidence interval. Any comparisons not crossing the dotted line are statistically significant (p < 0.05). (**A**, **B**) Compression testing using sequential application of coverslips showed a steady decrease in axial and equatorial strain with age, indicating that lenses from older mice are stiffer. (**C**) Very old lenses from 30-month-old mice had increased resilience, calculated as the ratio of the pre-compression over post-compression axial diameter. Resilience for 30-month-old lenses was 98.8% ± 1.2% while resilience for younger lenses was ~94-96%.

Next, we examined the biomechanical properties of lenses between 2–30 months of age. We utilized the application of sequential coverslips to compress the lens and then measured axial compressive strain (negative) and equatorial expansion strain (positive). Strain is a dimensionless measurement of percent change, allowing direct comparison between age groups regardless of changes in lens volume. Similar to previous reports [37–41], we observe that there is a steady increase in lens stiffness with age, demonstrated by the decrease in axial and equatorial strain under compressive load (Figures 1, 4A and 4B). After compression, we measured lens resilience (recovery after load removal) by comparing the pre- and post-loading axial diameter of the lens. Between 2-24 months, the resilience of the lens is similar, recovering to 94–96% pre-loading diameter (Figure 4C). Surprisingly, at 30 months, the resilience of the lens is even better, recovering to 98.8% ± 1.2%, and some of the lenses recovered completely to their pre-loading axial diameter. This may be linked to the dramatic increase in nuclear size in 30-month-old lenses. The mouse lens nucleus is very stiff and resists compression. Alternatively, very old lenses do not compress as much as the young lenses so it is possible that the increase in resilience may be because the oldest lenses do not need to recover as much after load removal.

Detailed analysis of strain data revealed that at the lowest load (129.3mg), there is an increase in stiffness of the lens between 2 to 8 months of age (Supplementary Figures 2A and 3A). Interestingly, lenses from mice over 4 months of age do not exhibit increased stiffness with age at this lowest load. This data suggests that the lens peripheral region, which is compressed by the lowest load, does not stiffen much with age. In contrast, at the highest load (1293mg), there was a general decrease in strain with age, suggesting that whole lens stiffness increased with age (Supplementary Figures 2B and 3B).

### Capsule thickness and fiber cell width do not increase after 4 months of age, but epithelial cell area increases slightly with age

**Figure 5:**
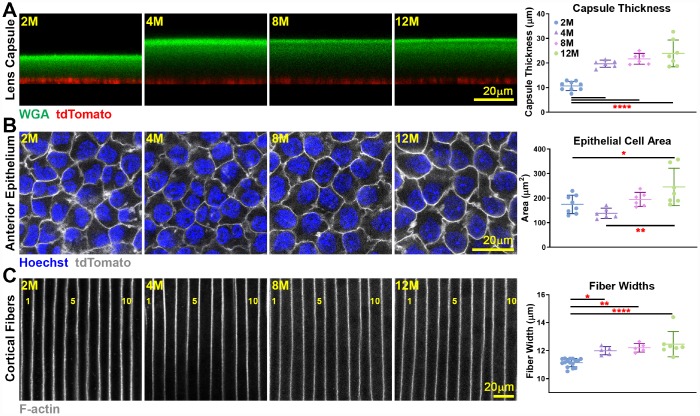
**Live lens measurements of capsule thickness and anterior epithelial cell area and fixed lens measurements of cortical fiber cell width.** Lines on the plots reflect mean ± SD of n = at least 6 lenses per age. Data from 2-month-old samples are reprinted from our previous publication [53]. *, *p*<0.05; **, *p*<0.01; ****, *p*<0.0001. (**A**) Lens capsule thickness increases between 2 months and older ages, but the thickness is unchanged after 4 months of age. WGA (lens capsule) is shown in green, and tdTomato signal (basal surface of anterior epithelial cells) is shown in red. (**B**) Anterior epithelial cell area is increased between 2 and 4 months and 12 months of age. Cell nuclei (Hoechst) is shown in blue, and tdTomato signal (lateral membrane of anterior epithelial cells) is shown in grayscale. (**C**) Cortical fiber cell width increases between 2-month-old and older lenses, but there is no increase in fiber cell width after 4 months of age. The fiber cells are numbered showing 11 full-width cells in the 2-month-old lens and 10 full-width cells in lenses that were 4 months and older. These measurements show that although mouse lenses continue to increase in size with age, capsule thickness and fiber cell size only increase until about 4 months of age. There is a mild increase in epithelial cell size up to 12 months of age.

We conducted a detailed study of the lens capsule and cell size in lenses from 2–12 months old mice. Utilizing WGA staining for the lens capsule and tdTomato+-labelled epithelial cell [53], we were able to measure capsule thickness in live lenses (Figure 5A). The lens capsule increased in thickness between 2 to 4 months of age but did not change in thickness beyond 4 months of age. Live lens imaging of anterior epithelial cells also revealed a small increase in epithelial cell area between young 2- and 4-month old and older 12-month-old lenses (Figure 5B). The small change in anterior epithelial cell size likely reflects an increase in lens volume accompanied by a mild decrease in the number of quiescent anterior epithelial cells with age [54]. We also measured cortical fiber cell width in fixed lenses and observed a slight increase in fiber cell width between 2- and 4-month-old lenses, but no further changes in cortical fiber width after 4 months of age (Figure 5C). This indicates that newly formed fiber cells remain at a constant width after 4 months of age. Since the lens continues to grow after 4 months without an increase in fiber cell width, the number of cells in newly added outer shells would increase to fill the larger lens circumference, and we would also expect increased axial length of fibers in aging lenses to stretch from the anterior to posterior poles.

### Anterior cataracts, cortical haziness and ring cataracts are observed in lenses from mice older than 12 months

**Figure 6:**
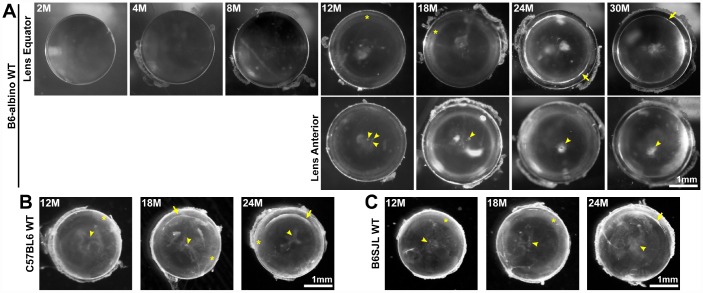
**Lens images (top down view) from mice between 2-30 months of age in various B6 wild-type backgrounds.** (**A**) B6-albino wild-type mice have clear lenses up to 8 months of age and develop small anterior opacities (arrowheads) by 12 months of age. Lenses from mice between 12-18 months develop cortical haziness (asterisks). Old lenses from mice between 24-30 months display ring cataracts (arrows) with a clear periphery and translucent, but not transparent, central regions. (**B**, **C**) Similar to B6-albino wild-type lenses, C57BL6 and B6SJL wild-type lenses also develop anterior opacities (arrowheads), cortical haziness (asterisks) and ring cataracts (arrows) at the same age as B6-albino wild-type mice. These images reveal that aged mouse lenses in the B6 genetic background develop cataracts around 12 months of age at the anterior pole and the lens cortex (haziness and ring opacity). Scale bars, 1mm.

Maintaining life-long transparency is essential for the function of the lens. We imaged freshly dissected lenses from 2-30-month-old mice, and determined that after 12 months of age, lenses very often developed anterior punctate cataracts (Figure 6A, arrowheads) and opacity in the lens cortex (Figure 6A, asterisks). The mild cortical haziness becomes a distinct ring in lenses by 24 months (Figure 6A, arrows). While the peripheral region of the lens outside the ring remained transparent, the inner portion of old lenses was translucent, but not transparent. The ring cataract causes a band of optical discontinuity that is more apparent when viewing the lens from the side (Figure 1, yellow arrowheads).

The mice we initially examined were in the B6-albino background from Jackson lab. Though there have been previous reports that mice in the C57BL6 genetic background develop cataracts with age [10, 12–15, 19, 20], these reports did not investigate the timing and location of cataracts in detail. Therefore, we also examined mouse lenses in two other B6 wild-type mouse lines to determine whether the timing of cataract occurrence and lens opacity locations were similar to the B6-albino wild-type mice. We found that B6 wild-type lenses from C57BL6 mice from Charles River and B6SJL mice from Jackson Lab also develop anterior opacities, cortical haziness and ring cataracts at approximately the same age as lenses from B6-albino wild-type mice (Figures 6B and 6C).

Almost all lenses from wild-type mice in various B6 backgrounds over 12 months of age developed anterior cataracts, cortical haziness or ring cataracts with varying degrees of severity (Table 1). There were no lenses from mice over 12 months of age without cataracts, and most aged lenses had multiple areas of opacity. In 12-month-old lenses, ~59% had anterior cataracts, ~85% had cortical haziness and ~3% of lenses were clear. In 18-month-old lenses, ~70% had anterior cataracts, ~87% had cortical haziness and ~10% had ring cataracts. In 24-month-old lenses, ~92% of lenses had anterior cataracts, ~38% had cortical haziness and 75% of lenses had a ring cataract. In 30-month-old lenses, all lenses had anterior and ring cataracts.

**Table 1:** Cataract phenotypes in WT mice of various B6 backgrounds.

			**Cataract location**		
	**Age**	**# of lenses**	**Anterior**	**Cortical**	**Ring**
**B6-albino**	<8M	28	0	0	0
	12M	26	12	22	0
	18M	18	10	17	0
	24M	16	12	6	12
	30M	12	12	0	12
**C57BL6**	12M	4	4	3	0
	17-18M	5	5	2	3
	24-25M	5	5	0	5
**B6SJL**	12M	4	4	4	0
	18M	7	6	7	0
	24M	5	5	4	1
	30-32M	4	4	0	4

Analysis of lens opacities in WT mice in various B6 backgrounds between 2-30 months of age. Most aged lenses (12+ months) displayed anterior cataracts, cortical haziness or ring cataracts, and many lenses had multiple opacities in different locations of the lens. Only one lens from a B6-albino 12-month-old mouse was clear and did not have obvious cataracts.

### Anterior cataracts in old lenses are due to incomplete suture closure and detachment of anterior epithelial cells from the underlying fiber cells

**Figure 7:**
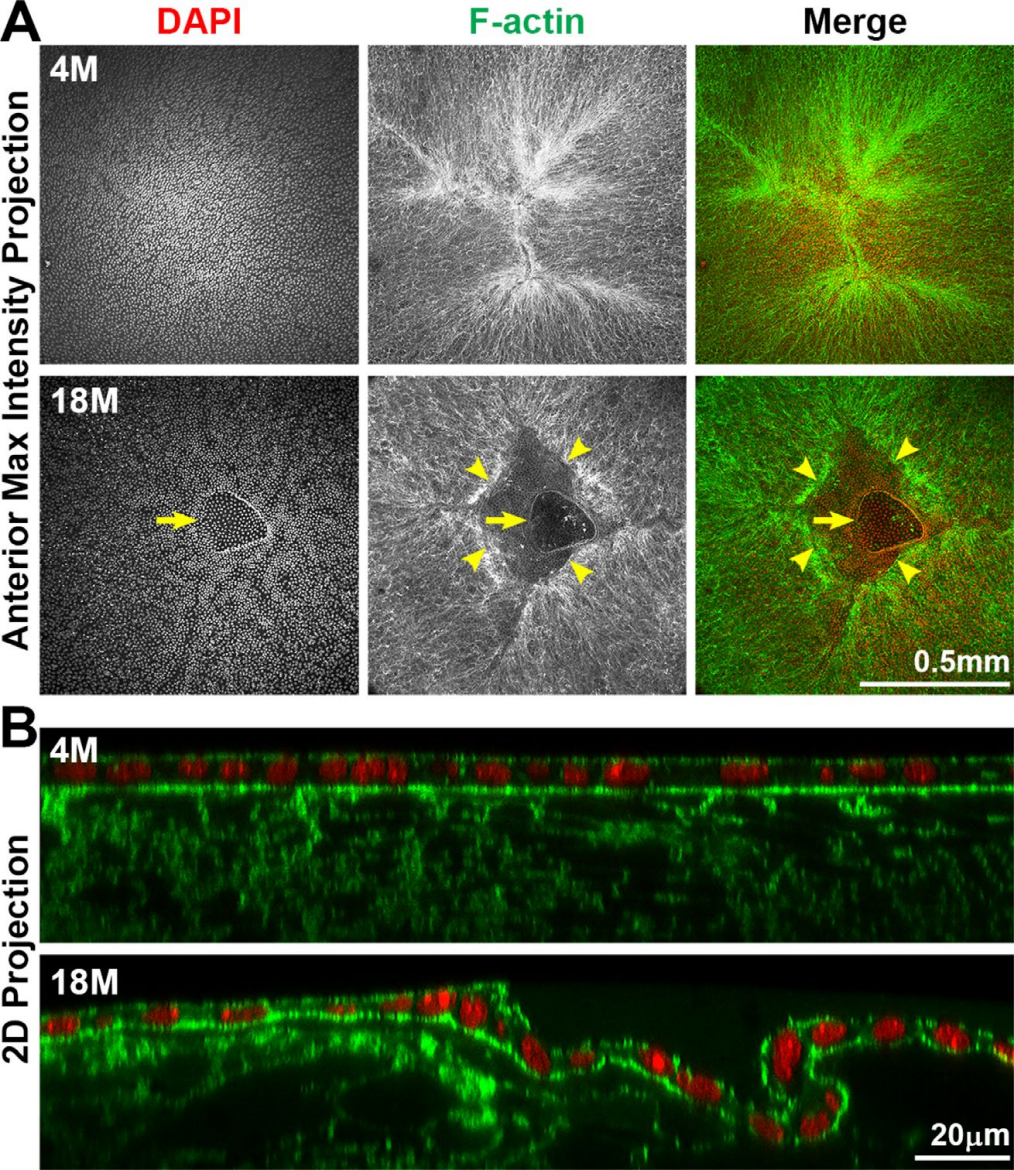
**Whole lens staining for F-actin (phalloidin, green) and nuclei (DAPI, red) in 4-month-old and 18-month-old lenses.** (**A**) The maximum intensity projection of the anterior lens epithelium and underlying fibers in the 4-month-old lens shows evenly distributed epithelial cell nuclei (DAPI) with a normal branched Y-suture (F-actin) under the epithelial cells. In contrast, there is an obvious defect at the apex of the 18-month-old lens with abnormal distribution of epithelial cell nuclei (DAPI, arrows) and a gap in the anterior suture (F-actin, arrowheads). (**B**) A 2D YZ projection of the 3D reconstruction of a Z-stack through the anterior epithelium and underlying fiber cells in the 4-month-old lens reveals tight adhesion of the anterior epithelial and fibers. In the 18-month-old lens near the fiber cell defect, the anterior epithelial cell layer is wrinkled and is depressed into the gap of the Y-suture. Although there was a defect in the epithelial cell sheet organization, there was no evidence of multilayered epithelial cells or abnormal epithelial cell proliferation in the 18-month-old lens. These results reveal that anterior cataracts in 18-month-old lenses are correlated with detachment and wrinkling of the anterior epithelial cells from the underlying Y-suture formed by fiber cells. Scale bars, 0.5mm in A and 20μm in B.

To determine the cause of anterior cataracts, we fixed lenses and performed whole lens staining and imaging of the lens anterior. We used phalloidin staining to determine whether there were changes in F-actin organization in anterior epithelial and fiber cells. Previous studies had suggested anterior subcapsular opacities were due to abnormal epithelial cells undergoing epithelial-to-mesenchymal transition (EMT) [55–59]. Surprisingly, we found that anterior cataracts in 18-month-old lenses (Figure 6, arrowheads) were correlated with incomplete closure of the anterior suture (Figure 7A). In younger lenses, as expected, elongating lens fiber cell tips meet at the anterior and posterior poles to form a Y-shaped suture (Figure 7A and Supplementary Figure 1A) [60]. The fiber cell tips forming the anterior suture are directly below and attached to the apical surface of the anterior epithelial cells [60]. Compared to the tight adhesion of anterior epithelial cells and the underlying lens fibers at the suture location in the 4-month-old lens, the entire epithelial sheet appears wrinkled and collapsed into the gap of the anterior suture, and the epithelial cells appear to be partially detached from the underlying fiber cells in the 18-month-old lens (Figure 7B). The location of this morphological defect in the lens epithelium is directly correlated with the presence of anterior subcapsular cataracts observed in lenses from mice over 12 months of age. Yet, despite detachment from the underlying fibers, anterior epithelial cells of the 18-month-old lens remained in a monolayer with no evidence of abnormal proliferation.

### Ring cataract in old lenses is linked to abnormal compaction of differentiating fiber cells

**Figure 8:**
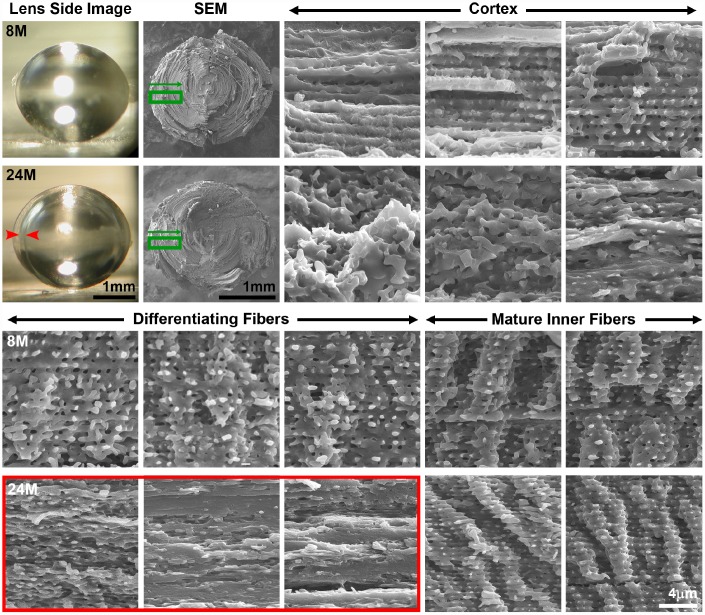
**Side view lens images and scanning electron microscopy (SEM) at various depths in 8-month-old and 24-month-old lenses.** Boxed regions in green on the low magnification SEM image indicate the approximate location where high magnification images were obtained. Cortical, newly formed fiber cells are disorganized in the 24-month-old lens compared to orderly cortical fibers in the 8-month-old lens. Differentiating fiber cells in deeper cortex layers (~100–200μm from the surface) of the 24-month-old lens lack normal small protrusions and formed a distinct zone of compaction. The location of the zone of compaction is correlated with the ring opacity (red arrows, red box). Mature inner fiber cells (~200-400μm from the surface) are comparable between the 8- and 24-month-old lenses with large paddles and small protrusions. Scale bars, 1mm (lens picture and low magnification SEM) and 4μm (high magnification SEM).

To determine whether there are cell morphology changes associated with the ring cataract in lenses from mice older than 24 months of age (Figure 6, arrows), we performed SEM on dissected lens halves from 8 months and 24 months of age to visualize fiber cell profiles from the peripheral fibers in the cortex to the differentiating fibers in the inner cortex to the mature inner fibers (Figure 8 and Supplementary Figure 1B). In the 8-month-old lens, cortical fibers are organized with clear cell-cell boundaries. As these fibers differentiate, there is the appearance of small protrusions along the short sides of the fiber cells in the 8-month-old lens. After the fiber cells undergo final maturation in the 8-month-old lens, we observe the normal large paddles and small protrusions that form the complex interdigitations of lens fiber cells (Figure 8, Mature Inner Fibers). The images of fiber cells from 8-month-old lens at various stages of maturation (i.e., depth) are consistent with previous studies of fiber cell morphology [50, 61–66]. In contrast, in the 24-month-old lens, we observe disorganized cortical fiber cells, and the differentiating fibers of the old lens were compacted in the region corresponding to the ring cataract (Figure 8, red arrows and red box). Surprisingly, the inner mature fibers beyond the zone of compaction in the old lens have normal paddles and protrusions similar to cells in the young lens. Our data suggest that compaction of differentiating lens fibers within a narrow zone in the cortex is correlated with an optical discontinuity in the lens cortex leading to the ring cataract in the 24-month-old lens. This apparently differs significantly from the large, continuous areas of compaction normally seen in the nuclear fiber cells, which retain transparency in the aging lenses.

### Hexagonal fiber cell packing of fiber cells is disrupted with age

**Figure 9:**
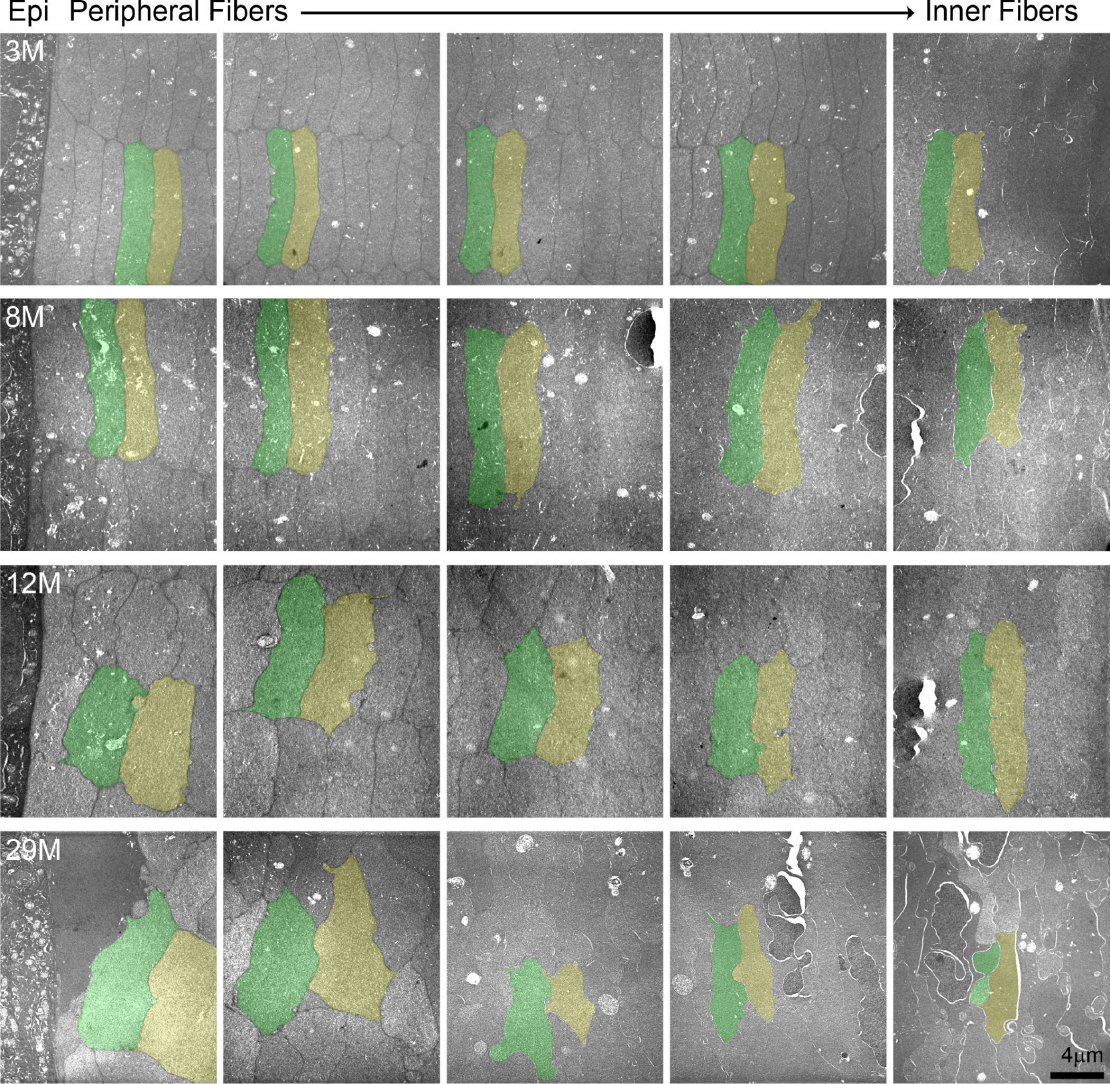
**Transmission electron microscopy (TEM) of lens cross sections at various depths in 3**–**29-month-old lenses.** Two neighboring cells in each panel are pseudo-colored green and yellow to show cell shape and size. In the 3- and 8-month-old lenses, fiber cells are hexagonal in shape and uniform in size from the periphery to the inner mature fiber cells. In the 12-month-old lens, the most peripheral fibers have lost their distinct hexagonal shape, but cells are still similarly sized between neighboring layers. In the 29-month-old lens, the cells have lost their characteristic hexagon cell shape and are highly variable in shape and size. There is also variability in electron density between neighboring cells in the 29-month-old lens with dark and light gray cells. Scale bar, 4μm.

Since we observed disorganization of the cortical fiber cells in the 24-month-old lens, we conducted TEM to determine the cell shape and organization of lenses from mice between 3–29 months of age (Figure 9). Cross sections through the lens periphery (Supplementary Figure 1B) include the epithelial cells on the left (labelled Epi), peripheral fiber cells and inner fiber cells on the right (Figure 9). The 3- and 8-month-old lens fibers are hexagonal in shape with uniform cell size. These young fiber cells are well aligned into neat rows and radial columns. In the 12-month-old lens, the newly formed fibers at the lens periphery in this older lens have lost their distinct hexagonal shape but are still mostly uniform in size and organized into radial columns spanning neighboring layers. In contrast, in the 29-month-old lens, the fiber cells have all lost their normal hexagonal shape, are highly variable in size with tortuous membrane contours and are no longer aligned into radial columns. The electron density of the fiber cell cytoplasm is also highly variable, such that neighboring cells with dark and light gray cytoplasm are visible. There is also a general disorganization of cells in this very old lens, likely due to the change in cell shape and size.

**Figure 10:**
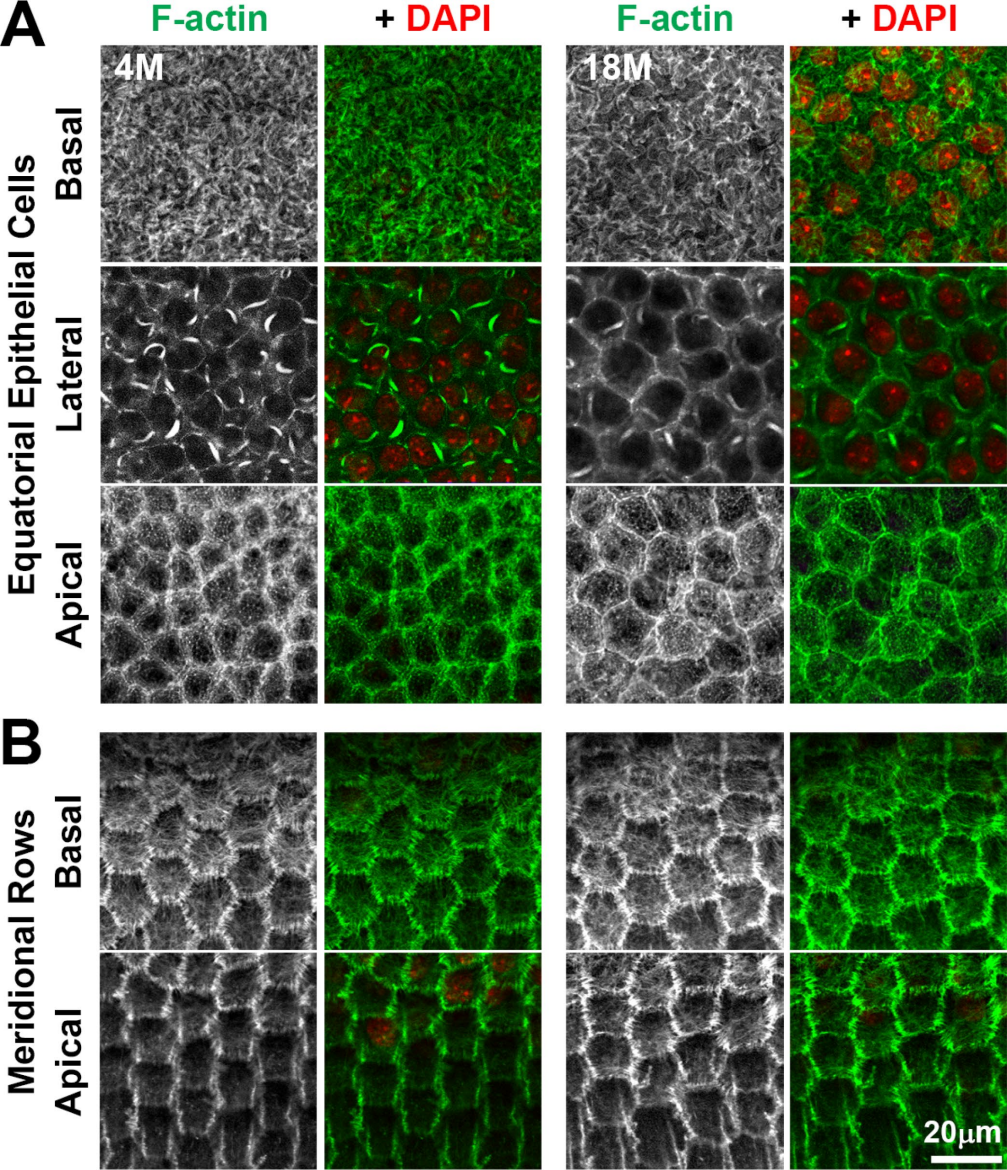
**Whole lens staining for F-actin (phalloidin, green) and nuclei (DAPI, red) in 4-month-old and 18-month-old lenses reveal that the actin cytoskeleton in epithelial cells and the formation of organized meridional rows of hexagonal equatorial epithelial cells is similar between 4-month-old and 18-month-old lenses.** (**A**) Single XY planes through anterior epithelial cells show similar F-actin staining and organization of equatorial epithelial cells between 4-month-old and 18-month-old lenses. These cells have a network of basal F-actin, membrane-adjacent F-actin and sequestered actin bundles near the lateral membrane, and polygonal arrays on the apical surface. (**B**) Single XY planes through the meridional rows at the lens equator reveals organized hexagonally-shaped epithelial cells with normal membrane-adjacent F-actin networks and a basal meshwork of F-actin in the 4-month-old and 18-month-old lenses. These data reveal that fiber cell shape changes and disorganization in older lenses is not due to altered shape or misalignment of equatorial epithelial cells. Scale bar, 20μm.

Hexagonal shape, packing and radial alignment of fiber cells originates from the F-actin dependent morphogenesis of the equatorial epithelial cells into meridional rows before fiber cell elongation and migration [46, 47]. F-actin polymerization can be affected by the redox state of the cell during aging and pathology (reviewed in [67]), and oxidative stress is a possible cause for cataractogenesis [1]. Therefore, we compared the packing and alignment of the anterior and equatorial epithelial cells, as well as the F-actin cytoskeleton in young and old lenses (Figure 10). We observed no obvious difference in the F-actin cytoskeleton of anterior epithelial cells between the 4-month-old and 18-month-old lenses (Figure 10A). Consistent with previous work [68–75], we observed F-actin stress fibers and lamellipodia on the basal surface, sequestered actin bundles (SABs) and cortical F-actin near the lateral membrane and polygonal arrays on the apical surface of the anterior epithelial cells. At the lens equator, we observed organized hexagonal-shaped epithelial cells that are lined up in neat rows in the 4-month-old and 18-month-old lenses (Figure 10B). F-actin is enriched at the cell membrane with a basal F-actin meshwork, similar to previous reports [47, 69]. Thus, these data indicate that the F-actin cytoskeleton is not significantly altered with age in lens epithelial cells and that alterations in hexagonal cell shape in fiber cells of old lenses are unlikely to arise from a defect in equatorial epithelial cell shape, alignment and organization.

### Gradient refractive index increases then plateaus with age

**Figure 11:**
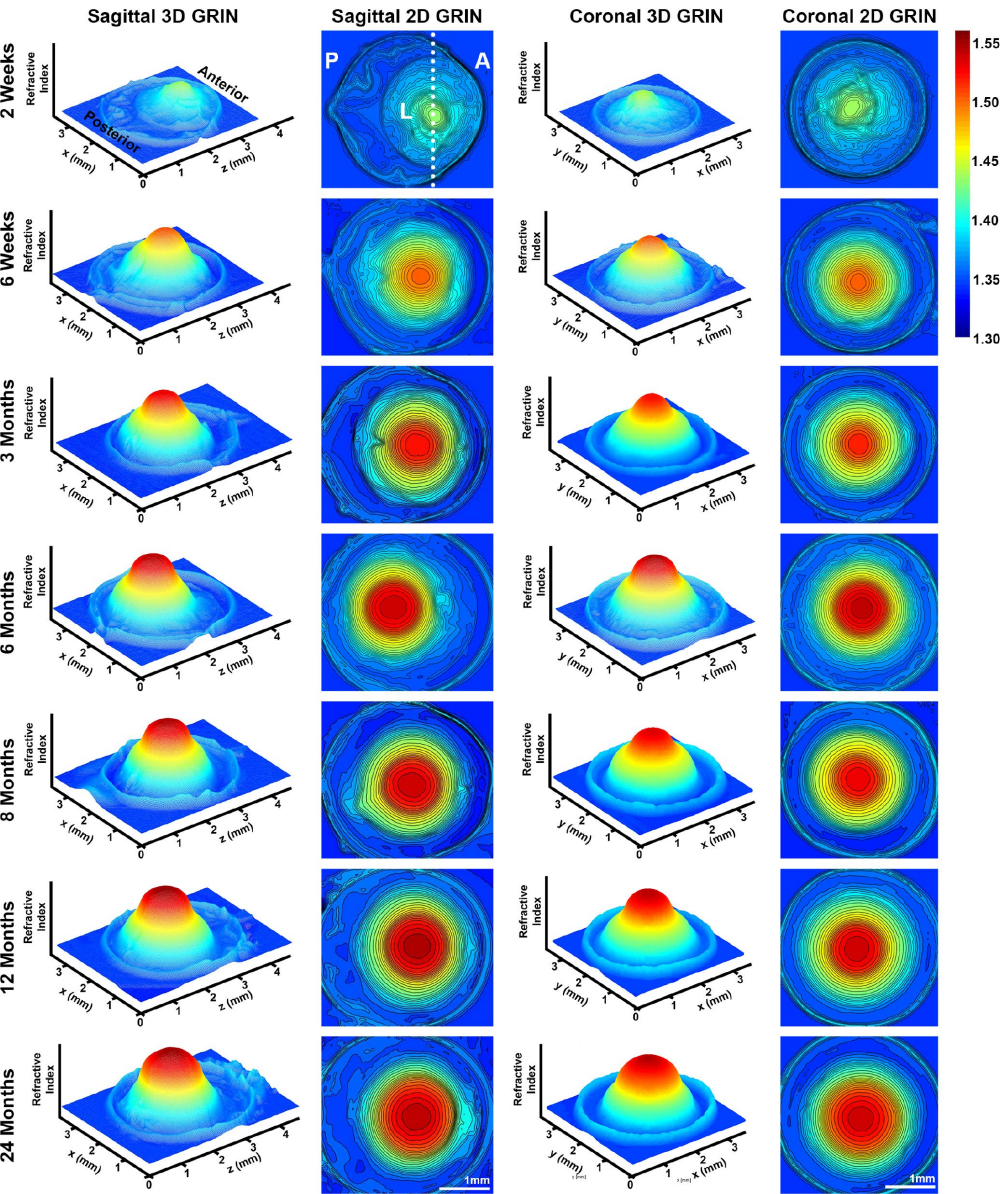
**3D mesh and 2D contour plots of the gradient of refractive index (GRIN) in whole eyes from mice between 2 weeks to 24 months of age.** Plots are through the mid-sagittal plane and the mid-coronal plane passing through each central lens nucleus. The anterior of the eye (A), the posterior of the eye (P) and the lens (L) are marked on the mid-sagittal views of the 2-week-old eye. The dotted line through the 2D sagittal view of the 2-week-old eye represents the location of the mid-coronal 3D and 2D heat maps. All images are oriented in the same direction. Colors reflect the magnitude of refractive index from low refractive index in dark blue (1.30) to high refractive index in dark red (1.55). The areas with highest refractive index are the lens. Mouse lens GRIN profiles are two-tiered with a ring of indentation (bright yellow) clearly seen in the 3D mesh plots. There is a cap region of high refractive index (red and orange) and a bottom region (yellow and green). There is an increase in the size of the cap region with age. These data show that GRIN in mouse lens develops by 2 weeks of age, and there is a rapid increase and plateau of maximum refractive index at the center of the lens with age.

**Figure 12:**
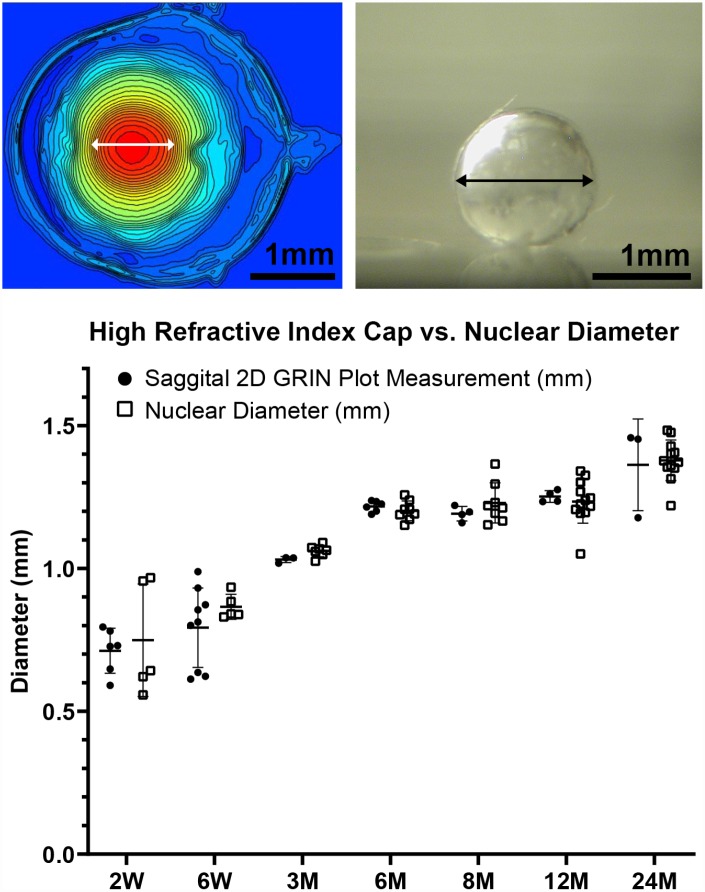
**A comparison of the diameter of the cap region of high refractive index and the diameter of the extracted lens nucleus.** The images show a representative mid-sagittal 2D contour plot and a representative lens nucleus with double-headed arrows indicating measured diameters. The graph compares the cap diameter in the sagittal 2D GRIN plot to the diameter of the lens nucleus. Lines on the plots reflect mean ± SD of n = at least 3 lenses from different mice per age. There was no statistically significant difference between the cap and nucleus diameters indicating that the area of high refractive index is directly correlated with the hard and compact lens nucleus. Scale bars, 1mm.

A high refractive index is necessary for the fine focusing power of the lens to transmit a sharp image onto the retina. We measured the refractive index in whole mouse eyes and generated 3D mesh plots and 2D contour plots of refractive index distribution in both the mid-sagittal plane and the mid-coronal plane passing through each central lens nucleus. Representative plots are shown for mouse eyes from 2 weeks to 24 months of age (Figure 11). The magnitude of refractive index is indicated using colors ranging from low refractive index in dark blue (1.30) to high refractive index in dark red (1.55). Mouse lenses occupy around three quarters of the space inside the eye, and the lens is easily distinguished in the plots as the region with highest refractive index. Lenses at all ages have highest magnitude of refractive index at the center, which plateaus over a limited region and decreases progressively toward the lens cortex, forming a Gradient Refractive Index (GRIN) in the lens. The GRIN profile is two-tiered with a ring of indentation that be clearly seen from the 3D mesh plot (bright yellow region). This indentation area separates the GRIN profile into two distinct regions: a cap (in red and orange) region and a bottom (in yellow and green) region. We measured the diameter of the cap area in mid-sagittal 2D contour plots and compared that with the diameter of the isolated lens nucleus (Figure 12). The diameters of the cap area and the lens nucleus are very similar, with no statistically significant differences, suggesting that the highly compact lens nucleus is responsible for the high refractive index cap region on the 3D GRIN mesh plots. The bottom region of the GRIN profile, therefore, corresponds to the lens cortex.

**Figure 13:**
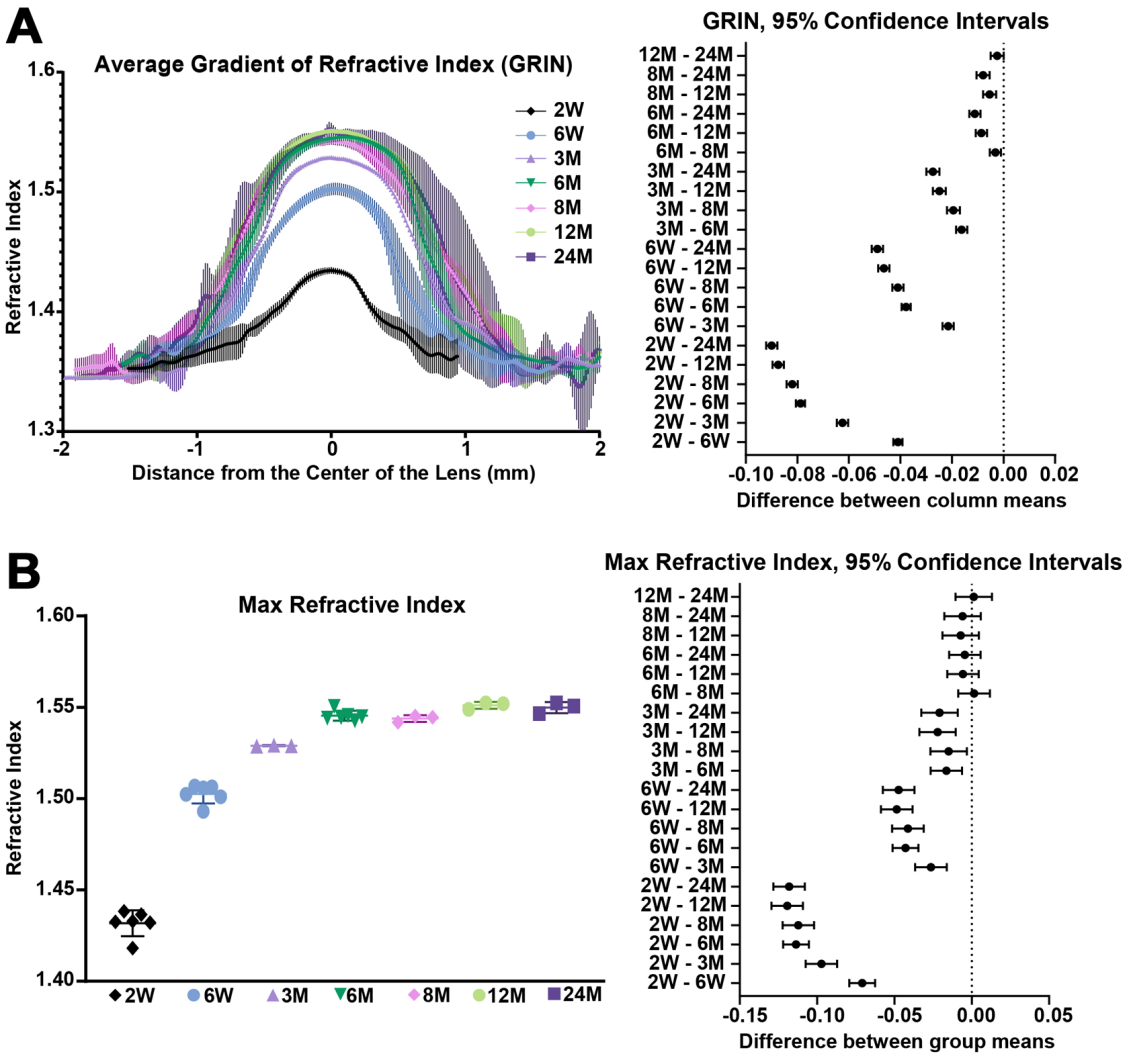
**Average GRIN profiles along the visual axis and maximum refractive index in lenses from mice between 2 weeks to 24 months of age.** Lines on the plots reflect mean ± SD of n = at least 3 lenses from different mice per age. The graph next to the data plots shows the 95% confidence interval. Any comparisons not crossing the dotted line are statistically significant (p < 0.05). (**A**) Average GRIN profiles increased in magnitude until about 6 months of age and then remained relatively unchanged with age. There is a statistically significant difference between profiles of different ages, except between the 12 months and 24 months profiles. (**B**) Max refractive index is ~1.55 in mouse lenses. Maximum refractive index rapidly increases until 6 months of age and then remains steady after 6 months of age.

We also extracted and plotted the average GRIN profile of lenses through the visual axis (Figure 13A). The GRIN profiles of lenses from 2-week-old mice have relatively low refractive index, and with age, especially between the period from 2 weeks to 6 weeks, the GRIN increases over the entire lens. The GRIN profile shows a central plateau region that increases in magnitude with age until about 6 months of age, after which the magnitude of the central plateau region remains relatively unchanged. GRIN profiles are statistically different between all age groups except between 12 months and 24 months. This suggests that there was little or no change in the GRIN profile after 12 months of age. This average GRIN profile obscures the indentation region separating the cap and bottom regions of the profile that are visible in the 3D mesh plots. We plotted representative 2D GRIN profiles from lenses at various ages (Supplementary Figure 4) with pairs of arrowheads to indicate the indentation at the edge of cap regions. From the GRIN profiles, we extracted and compared the max refractive index (Figure 13B). The maximum refractive index is ~1.55 and is reached at 6 months of age with no additional increase with age. Thus, while the region with high refractive index expands with age, the maximum refractive index does not increase significantly.

## DISCUSSION

**Figure 14:**
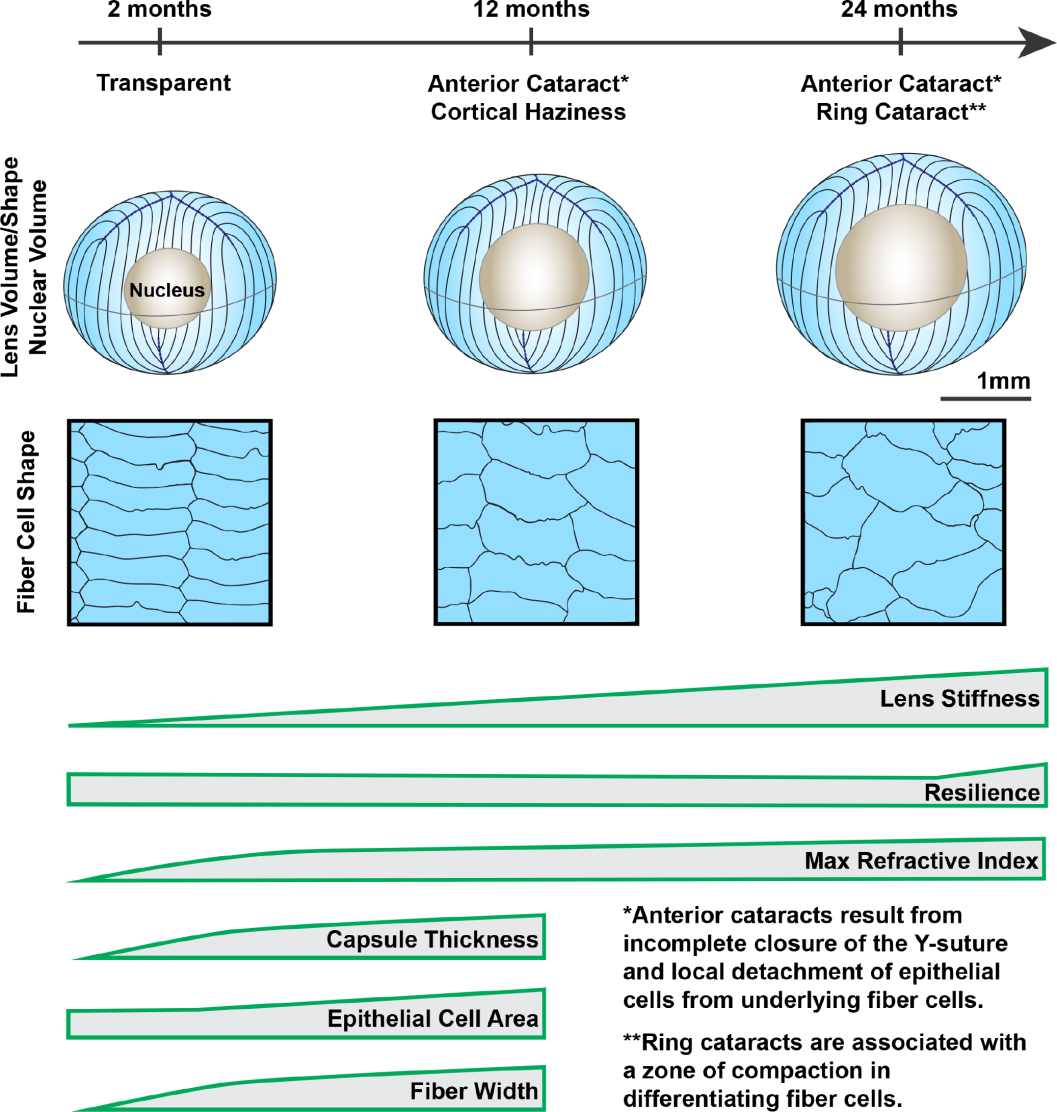
**Wild-type mouse lenses in the B6 genetic background showed increased volume, nucleus size and overall stiffness, changes in cell morphology and microstructure along with appearance of anterior, cortical and ring cataracts with age.** Lens volume and nucleus volume increase steadily with age. The shape and size of lens fiber cells become more disorganized in aged lenses. With age, mouse lenses develop anterior and cortical cataracts. Anterior cataracts are correlated with detachment of the anterior epithelial cells from the underlying fiber cells. Cortical ring opacities in the aged lenses are due to a zone of compaction in the cortical fiber cells leading to an optical discontinuity. While there is a steady increase in lens stiffness with age, resilience, or lens elasticity, is only increased in very old lenses. The maximum refractive index at the center of the lens (nucleus) increases rapidly until 6 months of age and reaches a plateau at 6 months. Lens capsule thickness and fiber cell width remain steady after 4 months of age, while epithelial cell area increases slightly between 4 and 12 months of age. Cartoons not all drawn to scale.

Our comprehensive study of aging in wild-type mouse lenses in the B6 genetic background showed increased stiffness along with appearance of anterior, cortical and ring cataracts with age (Figure 14). These data indicate that functional properties of wild-type mouse lenses change significantly with age and may be a suitable model to dissect the cellular and molecular mechanisms of age-related cataracts and changes in lens stiffness. The average life span of wild-type mice in the B6 genetic background is 26–29 months [76, 77]. Thus, in a relatively short period of time, we have been able to carry out this aging study. Our data shows that similar age-related cataracts are present in at least 3 different wild-type mouse lines in the B6 background, demonstrating that the source of the mice (Jackson Lab or Charles River) and the environment (Scripps Research Institute or Boston University) does not affect the cataract phenotype. This suggests that there may be a universal mechanism underlying the age-related opacities seen in the B6 wild-type mouse lenses.

While the changes in lens opacity are similar between the different B6 wild-type mouse lines, we do observe slight difference in lens size and stiffness with age. Though all lenses increase in size with age, our lens imaging data showed that B6-albino wild-type lenses are slightly larger than age-matched C57BL6 and B6SJL wild-type lenses (Figure 6). The B6-albino lenses were also softer than age-matched C57BL6 wild-type lenses at 2–8 months of age [41]. A more detailed study of lens biomechanics and morphometrics in different wild-type mouse lines and at different ages would be required to determine the exact differences between the slightly different B6 genetic backgrounds. These differences highlight the importance of using control mice, preferably littermates, of the same genetic background for comparison in all lens studies as subtle differences in lens properties can occur even in wild-type mice of the same genetic background.

### Mechanisms of age-related cataracts

Our data show that, in aged mouse lenses, anterior subcapsular cataracts arise due to detachment of the anterior epithelium from the underlying suture while the cortical ring cataracts are associated with an abnormal zone of compaction in the differentiating fiber cells. To our knowledge, this is the first report that age-related opacities in B6-albino wild- type lenses can be due to structural changes of the cells. These structural changes could themselves be caused by other better studied cataractogenesis mechanisms, such as oxidative stress, protein aggregation or disruption of lens fluid and ion homeostasis [1, 48, 78]. It is known that loss of both connexin 46 (α3) and connexin 50 (α8) and therefore gap junction coupling between fiber cells, in mouse lenses leads to incomplete anterior suture closure and severe cataracts [79]. Age-related loss of connexins, due to oxidative damage [78, 80], could lead to inadequate gap junction coupling and the formation of a suture gap in old lenses. The exact mechanism for suture disruptions with age require further study.

In human lenses, a barrier region has been observed at the interface between the lens cortex and the nucleus that forms during fiber cell maturation [81–83]. It is thought that the barrier limits antioxidant and metabolite movement into the lens and waste out of the lens leading to increased stiffness and decreased transparency with age [83]. Though the barrier observed in human lenses has not been reported in mouse lenses, previous reports have shown a diffusion barrier in rat lenses [84] and bovine lenses [85, 86]. In bovine lenses, the barrier region is in the approximate region of fiber cell compression (relative to lens size) [85, 86] that we observe in mouse lenses with advanced age. The exact structural and molecular basis for the barrier region remains unclear. In rat lenses [84], the diffusion barrier appears to be located near the site of membrane insertion for MP20, an abundant lens membrane protein. Our SEM data may suggest a possible structural alteration that leads to the formation of a diffusion barrier, in that the compaction of lens fiber cells could alter the fluid and ion outflow pathway. The avascular lens generates its own microcirculation system to allow transport of nutrients into and waste out of the tissue [87]. The outflow pathway is facilitated by a network of large micron-sized gap junction plaques in the differentiating lens fiber cells [88]. The disruption of gap junction plaques leads to decreased coupling between fiber cells [89], while loss of connexin proteins, due to genetic mutations or during aging, result in decreased gap junction coupling and cataracts [78, 80, 90–92]. Previous work also showed significantly decreased gap junction coupling in lenses from mice at 14 months of age as compared to those from mice between 2 and 6 months of age [78]. Further studies of connexins and gap junction plaque structures in the region of fiber cell compaction in the aging mouse lenses will be necessary to evaluate these mechanisms.

In our old lenses, the tissue inside the ring cataract appears hazy and translucent, rather than transparent. The hazy appearance may be due to compromised crystallin associations and organization in inner fiber cells due to decreased microcirculation in the old lens. While protein breakdown or aggregation may also lead to changes in transparency, the GRIN profile suggests that protein concentration is not significantly altered in the central region of the old lenses but shows greater variation in the cortex (Figure 13A). Overall lens volume does not increase significantly after 18 months of age. This could be due to the reduction of the growth rate or the fact that as even if new fiber cells were added at a constant rate, newly differentiated cells in older, and hence, larger, lenses would be increasingly stretched to cover a larger surface area and consequently be much thinner and appear more compact. Our measurements indicate that lens capsule thickness plateaus at 4 months of age and remains a steady thickness thereafter, and this contrasts with the continuous increase in lens volume up to 18 months of age. Thus, the biomechanical properties of the lens capsule may restrict, to some extent, the amount of available space for addition of new cell layers and could impart a compressive force as new layers are continuously added. Any compression, however, would be of a low degree such that it would not increase the local protein concentration in the outermost cell layers as this would manifest in a localized rise in refractive index, which could cause scatter.

### Cell morphology in aged lenses

We were surprised to find that lens fiber cells lose their characteristic hexagonal cell shape in lenses from mice over 12 months of age, and that the fiber cell membrane contours appear less straight with age. In human lenses, the loss of hexagonal cell shape and the increase in tortuosity of fiber cells membranes has also been reported [93, 94]. The cell shape change in older lenses leads to abnormal packing of fibers cells causing a disruption of the neat rows of cells. On the other hand, the hexagonal packing of the equatorial epithelial cells appears to be unaffected in old lenses, indicating that fiber cell shape is altered during subsequent differentiation and elongation. The disordered fiber cell packing near the lens periphery does not appear to affect the transparency of the lens since the outer cortex of very old lenses is transparent. There is little data in the literature regarding lens fiber cell shape changes with age, due in part to difficulty in fixation and sectioning of mouse lenses [62]. More work needs to be done to understand how fiber cell shape is altered in very old lenses and whether there are obvious changes in the cytoskeleton and membrane structures with age.

### Increased lens stiffness with age

Increased stiffness in aging human lenses has been hypothesized to be caused by increased lens nucleus size and stiffness with age [21]. While our data shows increased nucleus size and overall tissue stiffness in aged mouse lenses, the correlation between nucleus size and lens stiffness is not clear. Between 24 and 30 months of age, mouse lens nuclei increase dramatically in size, but lens stiffness, while statistically different, is not greatly increased between 24- and 30-month-old lenses. Moreover, our recent study of lenses with altered F-actin-binding proteins showed that in mouse lenses, it is possible to have a softer lens with an enlarged and stiff lens nucleus [61]. Unlike the human lens nucleus, the mouse lens nucleus is not likely to contribute to overall tissue stiffness because mouse lenses do not change shape to accommodate and are much more spherical in shape than human lenses.

We also considered the possibility that the zone of compaction may affect the stiffness of aged lenses. We measured the equatorial expansion (strain) of the ring in lenses from 24- and 30-month-old mice (data not shown). A comparison of equatorial strain of the overall lens vs the strain of the lens materials within the ring opacity revealed no statistically significant difference, suggesting that the zone of compaction expands along with the rest of the tissue under compression. This is not surprising since the zone of compaction is a very small percentage of the overall lens and thus, would be unlikely to account for the significant increase in lens stiffness with age. More sensitive lens biomechanical measurements along with modeling of the tissue changes under compression would be required to confirm this hypothesis.

We explored other possible mechanisms for increased lens stiffness with age. Our detailed measurements of capsule thickness in aging lenses are consistent with increased thickness previously reported in mouse [95] and human lenses with age [96, 97]. In human lenses, the capsule thickens and stiffens with age, and thus, it has been suggested that the lens capsule plays a role in increased human lens stiffness with age [97]. In contrast, while the mouse lens capsule thickness increases between 2–4 months of age, the capsule thickness remains constant after 4 months while the lens continues to stiffen. Therefore, while the lens capsule thickness may influence increases in stiffness at younger ages up to 4 months, the contribution of the capsule to overall lens biomechanics after 4 months is unlikely to be due to capsular thickness. However, other properties in the capsule may change with age. In human lenses, capsular thickness peaks at the age of ~80 years, but capsule stiffness continue to increase with age [97]. This suggests that other alterations, chemical and/or structural may occur to the capsule matrix network with age. Of note, advance glycation end product levels increase with age in the lens [98], and an increase in advance glycation end products can cause the formation of covalent collagen crosslinks, which lead to increases in matrix stiffness [99]. Further work needs to be done to test whether mouse lens capsules have altered biomechanical properties with age and to examine the role the lens capsule plays in determining whole lens biomechanical properties.

Similar to previous studies in mouse and human lenses [54, 100–102], we observe increased epithelial cell area with age. We have recently shown that lens shape change requires epithelial cell expansion [53]. Therefore, it may be postulated that whole lens shape change under load is impaired in older lenses as epithelial cells are larger, flatter and have a decreased ability for further expansion. However, our findings show that epithelial cell area does not increase until 8 months of age. Thus, the increase in whole lens stiffness, at least between 2–8 months of age, is not due to a restriction in expansion ability of epithelial cell area. To our knowledge, we are the first to characterize fiber cell width changes with age, and our data shows that the increase in fiber cell width only occurs between 2 and 4 months of age with no further increases between 2–12 months. The age-dependent increase in peripheral fiber cell widths would not necessarily restrict whole lens shape change under load and therefore, cannot account for the continual increase in whole lens stiffness with age. Of note, a previous SEM study of adult mouse lens fiber reveals an average fiber cell width of 5.45μm in the mid-cortex region [43], a much deeper layer in the lens than our measurements. The difference in fiber cell width from the outermost cortex (our measurements of 11–12μm) with the differentiating cells of the mid-cortex (5–6μm) suggests that there is remodeling and compaction of the lens fibers during differentiation.

Finally, in very old and stiff mouse lenses, the misalignment of the lens fiber cells does not alter lens stiffness, suggesting that hexagonal fiber cell alignment does not contribute significantly to overall lens biomechanical properties. Our previous work has indicated that the intercellular interdigitations of mature fiber cells regulated by the actin cytoskeleton may be more important for lens stiffness [50]. The importance of fiber cell alignment in overall lens stiffness still needs to be tested via high resolution imaging of live lenses during compression combined with mathematical modeling of the distribution of load on organized vs. disorganized lens fibers.

### Lens resilience

Elasticity and the ability to change shape during accommodation are essential for focusing on near objects in human lenses. The accommodative ability of human lenses decreases with age along with a sharp increase in stiffness, and presumably a decrease in elasticity [21–26]. We used resilience (recovery after load removal) calculations as a parameter to reflect elasticity. Our data reveals that mouse lens resilience hovers around 95% for all ages tested, except 30-month-old lenses where resilience is close to 99%. This may be due in part to the abnormally large nucleus in 30-month-old lenses that resists compression, similar to increased stiffness and size of the human lens nucleus with age. Our previous data suggests that resilience in mouse lenses depends on the recovery of the anterior Y-suture that opens up under coverslip compression [53]. Further confocal microscopy studies of very old lenses under compression would be needed to understand the impact of the Y-suture on resilience with age. We also considered the impact of the zone of compaction on lens resilience. However, due to the location of the zone of compaction in the inner cortex of the lens and the very thin region of abnormal fiber cells, it is unlikely that this zone would affect the resilience of the lens.

### Gradient refractive index in murine lenses

GRIN is a distinct characteristic of lens that helps correct spherical aberrations, and lenses from all species that have been measured, so far, have a GRIN profile (reviewed in [103]). The maximum refractive index value found in adult mouse lens (1.552± 0.006) is much higher than that of a human or a porcine lens, which are around 1.40 and 1.42, respectively [104, 105], and is slightly lower than that of fish lenses (1.57) [106]. Our data shows that the highest refractive index is reached in mouse lenses at 6 months of age and after that only the area of maximum refractive index increases with age. The shape of the GRIN profile varies between different species and changes during both the growth and aging processes. The GRIN profile of a mouse lens has a rounder central region than that of a human lens, which has a relatively constant index value at the center [103, 104, 107]. The area of highest refractive index in the mouse lens is correlated with the hard and compact nucleus (Figure 12). It is not clear whether nuclear compaction is required for high refractive index in the lens, though the region of highest refractive index in mouse and human lenses is correlated with the location of the nucleus [21, 52, 103]. In fish lenses, however, nuclear compaction does not seem to be required for high refractive index [106, 108, 109]. Alternatively, a high refractive index in the center of the lens could simply be a result of high protein concentrations. The central index reaches a maximum in lenses from some species where proteins in the nucleus are tightly packed because the cytoplasm is composed of almost pure protein [103]. During lens fiber cell maturation, cellular organelles are eliminated, leaving mature fibers without the ability to initiate de novo protein synthesis [42, 110]. Therefore, for the cytoplasm of the nuclear fibers to have high levels of protein, there could be dehydration of the nucleus to decrease free water content in nuclear cells [111], possibly through increased bound water due to post-translational modifications and aggregation of lens proteins [112]. To validate this theory, proteomic and water content analysis of nuclei from young and old lenses would be needed to compare the differences in nuclear protein content and composition with age and how that correlates with the increase in GRIN. Another intriguing possibility is enhanced transport of proteins from peripheral fibers into nuclear fibers, which has been suggested to occur in fish lens nuclei [113], but this mechanism has not been explored in mammalian lenses.

Mouse lens GRIN profiles also display a unique discontinuity (or indentation) in the contour of the profile in peripheral region of the lens, which can be observed in the 3D GRIN mesh plots and 2D GRIN profiles (Figures 11 and Supplementary Figure 2). In other words, the mouse GRIN profile has two radii of curvature; one in the central region and one in the peripheral region. Compared to the two-tiered mouse GRIN, the GRIN profile in most other species is parabolic without a central plateau [103]. The two-tiered GRIN profile in mouse lenses can be observed at 2 weeks of age, the youngest lenses that were measured in the present study. It is hypothesized that formation of GRIN profile may take place during gestational age, though the exact age for each species is unknown. A study of the fetal bovine lens shows that shape of GRIN profile is irregular until the middle of gestation when it rapidly takes on a second-order polynomial shape [114]. Future measurements with embryonic mouse lenses would help determine when the GRIN is established during development, and whether variations in rate or extent of crystallin synthesis during lens growth can explain the peripheral discontinuity in the GRIN profile.

### Concluding remarks

Our data reveals that not all properties of the lens continue to change with age and, at least in mouse lenses, most morphometric properties plateau by middle age (12–18 months). Though theoretically the lens continues to add more layers of fiber cells, there is no significant change in the lens size and shape after 18 months of age, suggesting there is a maximum size for the mouse lens. Nevertheless, lens stiffness continues to increase up to 30 months, indicating that increased lens size cannot explain the increase in lens stiffness. Thus, there must be other cellular and molecular mechanisms that contribute to the increased stiffness of very old lenses. This is just one example of the questions that still need to be investigated to identify the link between morphometric measurements and lens functions (refraction, transparency and stiffness).

Collectively, the increases in lens size and nucleus size are correlated with increase stiffness with age. The addition of new fiber cells at the lens periphery becomes disordered with age, but this does not appear to impact lens biomechanical properties. Cataracts in aged lenses can be due to cell structural abnormalities, including incomplete suture closure, collapse of the lens epithelial cell layer into the suture gap and loss of epithelial-fiber cell attachments and compaction of the cortical lens fiber cells forming a circumferential light scattering ring. GRIN is present in the lens from 2 weeks of age and continues to increase until about 6 months of age, after which the maximum refractive index remains stable. The increase in the area of highest refractive index at the center of the lens is directly correlated with the increase in lens nucleus size, suggesting nuclear compaction drives the maximum GRIN. Whether there is a common molecular mechanism that drives changes in all the measured parameters remains unknown, but further biochemical and cell morphology studies will be needed to determine how subcellular aging affects the whole tissue. Thus, our study provides a baseline for future studies of lens aging by providing quantitative measurements of key parameters and identifying common age-related changes in the overall tissue and in individual cells.

## MATERIALS AND METHODS

### Mice and lens images

All animal procedures were performed in accordance with recommendations in the ARVO Statement for the use of Ophthalmic and Vision Research, the Guide for the Care and Use of Laboratory Animals by the National Institutes of Health and under approved protocols from the Institutional Animal Care and Use Committees at The Scripps Research Institute, Indiana University and Boston University.

Wild-type [B6-albino (Jackson Laboratories strain 000058), C57BL6 (Charles River strain 027) and B6SJLF1/J (hereafter referred to as B6SJL, Jackson Laboratories strain 100012)] and Rosa26-tdTomato mice tandem dimer-Tomato (B6.129(Cg)-Gt(ROSA) (hereafter referred to as tdTomato, Jackson Laboratories strain 007676) in the C57BL/6J background between the ages of 2 weeks-30 months were used for experiments. B6-albino mice (B6(Cg)-*Tyr^c-2J^*/J) are wild-type C57BL/6J mice with a spontaneous mutation in the tyrosinase gene [115, 116]. The B6-albino mice were a generous gift from Dr. William E. Balch (The Scripps Research Institute).The tdTomato-positive (tdTomato+) mice, used for live imaging, express a tdTomato tandem dimer protein fused in frame to connexin 43 [117] (rendering it an inactive channel). Based on our previous study, tdTomato+ lenses are comparable in shape, size and stiffness to C57BL6/J wild-type lenses [53]. The tdTomato+ mice were maintained with a single copy of the transgene, and tdTomato-negative (tdTomato-) wild-type lenses were also used for experiments. Since previous studies revealed loss of specialized beaded intermediate filaments in the lens due to an endogenous mutation in the *Bfsp2/C*P49 gene [118–120] results in changes in lens transparency and stiffness [38, 40], genotyping for *Bfsp2/CP49* were performed by automated qPCR on tail snips (Transnetyx, Cordova, TN) to confirm that all mice were wild-type for Bfsp2/CP49. Male and female mice were used for experiments.

Mouse lenses were dissected immediately from freshly enucleated eyeballs in 1X Dulbecco’s phosphate buffered saline (DPBS, 14190, Thermo Fisher Scientific, Grand Island, New York). Images of freshly dissected lenses were captured using an Olympus SZ11 dissecting microscope with a digital camera (B6-albino wild-type) or an adapted Zeiss OpMi microscope with a D70 digital Nikon camera (C57BL6 and B6SJL wild-type). In side-view images, there is a band of mild opacity around the lens equator. This is due to lens dissection and severing of the attached zonular fibers from the lens capsule. This opacity is not a defect in the lenses.

### Lens biomechanical testing and morphometrics

Morphometrics and stiffness of freshly dissected B6-albino mouse lenses were tested in 1X DPBS at room temperature using sequential application of glass coverslips as previously described [37, 38, 41]. Briefly, lenses were compressed with a series of glass coverslips, and images were acquired using an Olympus SZ11 dissecting microscope with digital camera. After mechanical testing, the lens capsule was gently removed, and soft cortical fiber cells were dissociated by rolling the lens between gloved fingertips leaving a very hard and round lens nucleus (center region of the lens) for imaging. FIJI software was used to perform image analysis, and Excel and GraphPad Prism 8 were used to calculate and plot strain [ε = (d-d_0_)/d_0_, where ε is strain, d is the axial or equatorial diameter at a given load, and d_0_ is the corresponding axial or equatorial diameter at zero load], resilience (ratio between pre-compression axial diameter over post-compression axial diameter), lens volume (volume = 4/3×π×r_E_^2^×r_A_, where r_E_ is the equatorial radius and r_A_ is the axial radius), lens aspect ratio (ratio between axial and equatorial diameters), nuclear volume (volume = 4/3×π×r_N_^3^, where r_N_ is the radius of the lens nucleus) and nuclear fraction (ratio between the nuclear volume and the lens volume), respectively. Plots represent mean ± standard deviation. Two-way ANOVA with Tukey’s multiple comparisons test were used to determine statistical significance.

### Live lens imaging, capsule thickness and fiber cell width measurements

Imaging and analysis of live tdTomato+ and fixed tdTomato- wild-type lenses to determine lens capsule thickness, anterior epithelial cell shapes and fiber cell widths were performed as previously described [53]. Briefly, isolated lenses were stained with fluorescent CF640 dye conjugated to wheat germ agglutinin (WGA, 1:100, Biotium, Fremont, CA) and Hoechst 33342 (1:500, Biotium) in 1X PBS (137mM NaCl, 2.7mM KCl, 8.1mM Na_2_HPO_4_, 1.5mM KH_2_PO_4_; pH 8.1) for 15 minutes. Stained lenses were then transferred onto glass-bottomed culture dishes (10-mm microwell; MatTek, Ashland, MA) and immobilized anterior pole down, within 3-mm-diameter circular divots that were created, using a biopsy punch, in a thin layer of agarose (4% wt/vol in 1X PBS). Reactive oxygen species (ROS) are formed during confocal imaging of fluorescent probes in live tissues [121, 122]. Lenses were imaged in 3ml of 1X PBS containing 1.8 units of Oxyrase (Oxyrase, Mansfield, OH), an oxygen scavenger, to prevent ROS-related cell toxicity [122]. To determine fiber cell widths, tdTomato- wild-type lenses were fixed in 4% paraformaldehyde in 1X PBS for 30 minutes at room temperature. Following fixation, lenses were washed briefly in 1X PBS and placed in permeabilization/blocking solution for 30 minutes. Lenses were then incubated in permeabilization/ blocking buffer containing rhodamine-conjugated phalloidin (1:20, Thermo Fisher Scientific, Waltman, MA) and Hoechst 33342 (1:500) for 2 hours followed by orienting them on their sides in the 3-mm-diameter circular divots described above.

Images were acquired on a Zeiss 880 laser-scanning confocal microscopy using a 20x air Plan-Apo 0.8 NA objective (equatorial lens region) or a 40x oil Plan-Apo 1.4 NA objective (anterior lens region). Z-stack images were acquired using step sizes of 0.7μm (20X; objective) and 0.3μm (40X objective) and were processed using Zen (Zeiss) software.

Morphometric analysis was conducted using FIJI software as previously described [53]. To determine capsule thickness, line scan analysis was performed on XZ plane-view reconstructed images to obtain intensity distributions of WGA-stained capsules and tdTomato-labeled cell membranes. The distance between WGA and tdTomato peaks is indicative of capsule thickness. To determine epithelial cell areas, a region of interest (ROI) containing ~50–150 cells was traced using tdTomato-labeled cell membranes as a guide. The area occupied by the ROI was measured and the number of cells was determined by counting the number of Hoechst-stained nuclei within the ROI. Average epithelial cell area was calculated by dividing the ROI area by the total number of cells. To determine fiber cell widths of fixed lenses, the equatorial region of the lens was imaged by propping lenses on their sides within an agarose wedge. Images were acquired and fiber cell widths were measured ~10μm inward from the fulcrum using Distributed Deconvolution (Ddecon) ImageJ plugin and the Z-line predictive model [123]. Data for 2 month old lenses were reprinted from our previous publication [53].

### Phalloidin-staining of epithelial cells in whole lenses

Whole B6-albino lens fixation and staining was performed as previously described with a few modifications [47, 55]. Freshly enucleated eyes were collected from 4- and 18-month-old mice. An incision was made at the ora serata to allow fixative to penetrate the globe. Whole eyes were fixed in 4% paraformaldehyde in 1X PBS on ice for 30 minutes. Eyes were then washed and incubated overnight in 1X PBS at room temperature. After overnight incubation, lenses were microdissected out of the eye. Whole lenses were then permeablized with 0.3% triton X-100 in 1X PBS for 15 minutes at room temperature. After permeabilization, lenses were immersed in Vectashield mounting medium with DAPI (H-1200, Vector Laboratories Burlingame, CA) for 30 minutes at room temperature. Samples were then washed 3 times, 5 minutes per wash, with 1X PBS. Lenses were then incubated for 2 hours at room temperature with Alexa-488-phalloidin (1:10, A12379, ThermoFisher), and Hoechst 33342 (1:250, Biotium, Fremont, CA) diluted in 1X PBS. Samples were then washed 4 times, 5 minutes per wash, in 1X PBS. Stained lenses were stored in Vectashield mounting medium at 4°C until imaging. Lenses were imaged in FluoroDishes (World Precision Instruments, Sarasota, FL) with optical quality glass bottoms, and equatorial images were collected by immobilizing the tissue on its side using agarose wedges [55]. Images and z-stacks (10X objective with 4μm steps or 40X objective with 0.3μm steps) were collected in the lens anterior and equator using a Zeiss LSM780 confocal microscope. Volocity (Quorum, Puslinch, Ontario, Canada) software were used to create max intensity projections, and 2D YZ projections of the 3D reconstruction of a Z-stack through the anterior epithelium and underlying fiber cells were rendered in ZEN 2.5 software (Zeiss). Staining was repeated on at least 4 lenses from 2 different mice for each age, and representative data are shown.

### Scanning electron microscopy (SEM)

Eight- and 24-month-old B6-albino wild-type lenses were prepared for scanning electron microscopy (SEM) as previously described [50, 61, 62]. Briefly, freshly dissected lenses were fixed in 2.5% glutaraldehyde in 0.1M sodium cacodylate buffer (pH 7.3) at room temperature for 2–3 days. A sharp needle was used to bisect lenses along the visual axis, and lens halves were post-fixed in 1% aqueous OsO_4_ for 1 hour at room temperature. Samples were dehydrated using ethanol and were critical point dried in a Samdri-795 critical point dryer (Tousimis Inc., Rockville, MD). Lens halves were mounted and coated with gold/palladium in a Hummer 6.2 sputter coater (Anatech Inc., Union City, CA). Images were acquired with a JEOL 820 scanning electron microscope at 10 kV (JEOL, Tokyo, Japan). Using the lens nucleus as a reference, images from different regions of the lens were compared between samples (i.e., comparable regions were located based on measurements from the center outward). At least four lenses from 2 different mice were examined for each age, and representative images are shown.

### Transmission electron microscopy (TEM)

Three-, 8-, 12- and 29-month-old B6-albino wild-type lenses were prepared for transmission electron microscopy using a method modified from a previous study [124]. Freshly dissected lenses were fixed in 2.5% glutaraldehyde, 0.1M cacodylate buffer (pH 7.3), 50 mM L-lysine and 1% tannic acid for 2-4 days (2 days for younger lenses and 4 days for older lenses). Fixative was removed and exchanged with fresh fixative at after day 1 or day 3. Fixed samples were stored in 0.1M sodium cacodylate buffer (pH 7.3) at 4°C until processing for EM. Lenses were mounted onto the specimen holder with superglue with the equatorial surface facing up, and then 200μm-thick lens section in the cross orientation were collected using a vibratome. Lens sections were post-fixed in 1% aqueous OsO4 for 1 hour at room temperature. Sections were then rinsed in ddH_2_O and stained en bloc at 4°C overnight with 0.5% uranyl acetate in 0.15M NaCl. Samples were dehydrated through graded ethanol and propylene oxide and embedded in Polybed 812 resin (Electron Microscopy Sciences, Hartfield, PA). One-micron-thick sections from the samples were cut with a diamond knife and stained with 1% toluidine blue. A Zeiss light microscope was used to examine sections before thin sections (70–80nm thick) were cut with a diamond knife. Thin sections were stained with 5% uranyl acetate followed by Reynold’s lead citrate and examined in a JEOL 1200EX electron microscope at 80 kV (JEOL). At least 3 lenses from 3 different mice were examined at each age, and representative images are shown.

### X-ray talbot interferometry

Whole B6-albino mouse eyes were stored in Dulbecco's Modified Eagle Medium without phenol red (21063-029, ThermoFisher) with 2% penicillin/streptomycin (15-140-122, ThermoFisher) at room temperature before experiments. Three-dimensional refractive index distribution of mouse eyes were measured using X-ray Talbot interferometry, which is a synchrotron radiation-based phase contrast imaging modality [125–127]. Talbot interferometry is constructed at the bending magnet beamline BL20B2 at SPring-8. Experiments were conducted using monochromatic X-ray beam, fined tuned to 25 keV, which passes through a Si(111) double-crystal monochromator and two transmission gratings: a nickel phase grating (G1) and a gold absorption grating (G2). The pattern thicknesses of G1 and G2 are 4.35μm and 110μm, respectively. The pitch size of both gratings is 4.8μm, and the pattern size area is 50×50mm^2^. A scientific CMOS detector (ORCA Flash 4.0. Hamamatsu Photonics) is used to detect the Moiré fringe patterns generated by X-ray beam passing through the sample and two gratings. A piezo stage and a 5-step fringe-scan method was used to shift G2 for phase retrieval, and a phase shift image was integrated from differential phase images obtained from the scan. To calibrate the phase shifts, experimentally obtained values for five different solutions of known density [127, 128] were compared to theoretically derived values per pixel. Linear relationships were found over the range of tested concentrations. X-ray refractive index difference was determined from the phase shifts per pixel using equations described previously [127]. Protein concentration, determined from the X-ray refractive index difference, is linearly related to the refractive index of the lens [129]. The total number of projections for tomography was 900, and the time of measurement is 50 minutes for each eye. Refractive index values measured by X-ray Talbot interferometry were processed by MatLab software (2018a, MathWorks, Natick, MA) to generate 2D iso-indicial index contours and 3D meshed index profiles in the mid-sagittal plane of each mouse eye. Gradient of refractive index (GRIN) profiles were generated along the visual axis by MatLab, and the average and standard deviation were calculated in Excel and plotted in GraphPad Prism 8. One-way and two-way ANOVA with Tukey’s multiple comparisons test were used to determine statistical significance.

## Supplementary Material

Supplementary Figures
