# Supplementary material for: Age-related changes in eye lens biomechanics, morphology, refractive index and transparency
Source: Aging (Albany NY). 2019 Dec 16;11(24):12497–531. doi: 10.18632/aging.102584 (PMC6949082; doi:10.18632/aging.102584)
Supplement: Supplementary Figures [file aging-11-102584-s001..pdf]

SUPPLEMENTARY FIGURES

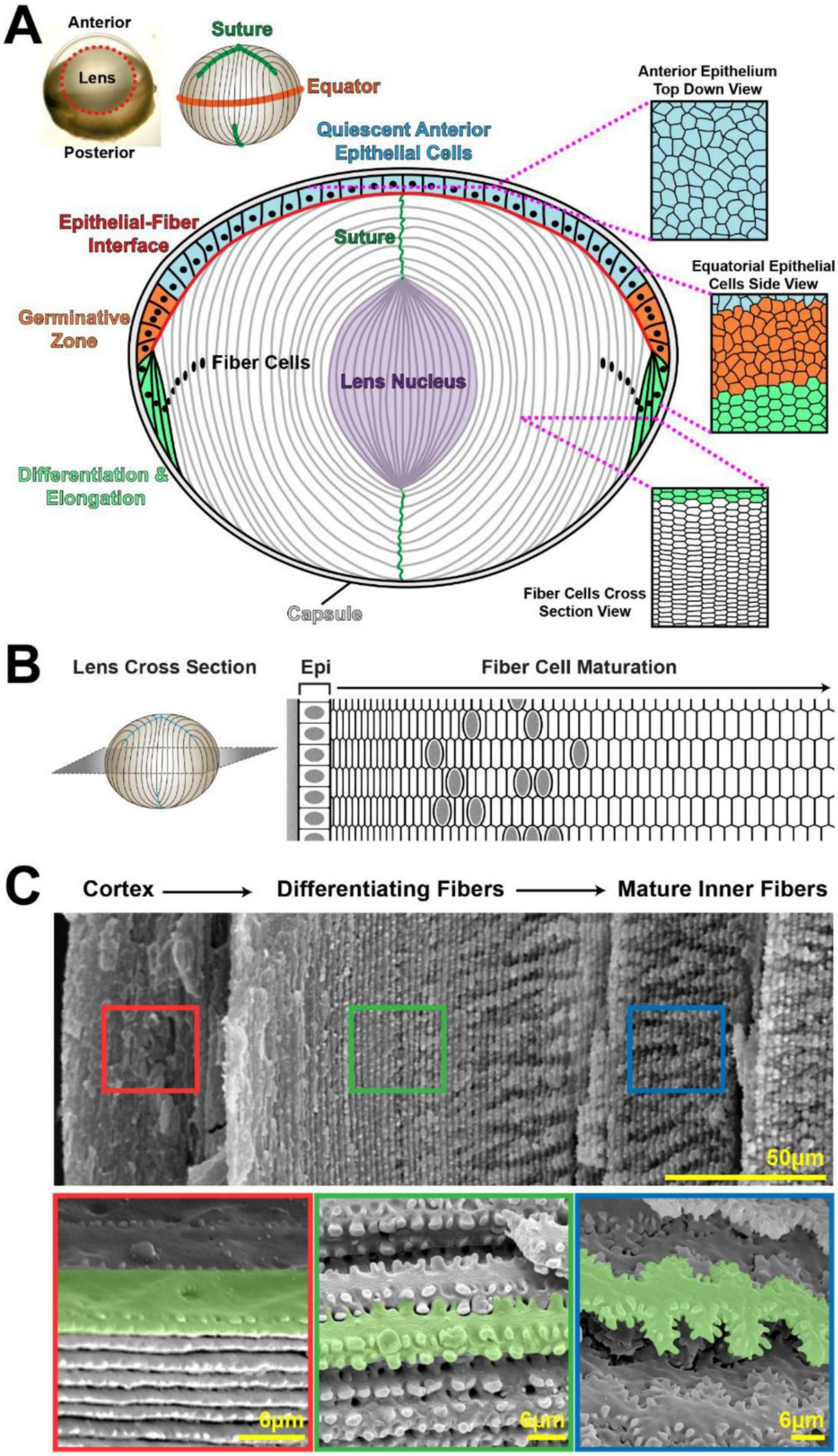

Supplementary Figure 1. Lens anatomy diagrams. (**A**) A cartoon (not drawn to scale) depicting a longitudinal section of the lens shows a monolayer of epithelial cells at the anterior surface (colored cells) and a bulk mass of elongated lens fibers (white cells) that extend from the anterior to posterior poles. The entire tissue is encapsulated by a thin collagenous basement membrane called the lens capsule. Anterior epithelial cells (blue) are quiescent and do not proliferate. These cells are cobblestone in shape, and their main function is to maintain the underlying fiber cells. Lifelong lens growth relies on the proliferation, migration and differentiation of equatorial epithelial cells (orange) in

the germinative zone. Equatorial epithelial cells undergo a remarkable morphogenesis to transform from randomly organized cuboidal cells (orange) into organized rows with hexagon cell shape (green). A cross section through the fiber cells reveals that the organization and hexagon cell shape of the equatorial epithelial cells are retained in fiber cells. New layers of lens fiber cells are continuous added onto previous generations of fibers, and the lens grows in concentric shells. Newly formed fiber cells elongate toward the anterior and posterior poles. The apical tips of newly formed fibers migrate along the apical surface of epithelial cells toward the anterior pole while the basal tip of the newly formed fibers migrate along the lens capsule toward the posterior pole. At each pole, the elongating fibers will detach from the epithelial cells or lens capsule and contact the elongating fiber from the other side of the lens forming the Y-suture. Differentiating fiber cells also undergo a maturation process and lose all of their organelles to allow for a clear light path. The mature fiber cells in the middle of the lens without cellular organelles have very low metabolism and little or no protein turnover. The lens nucleus is composed of tightly compacted fiber cells in the middle of the lens (purple). (**B**) Diagram of a lens equatorial cross-section (not drawn to scale, modified from [51]). Epithelial cells (Epi) are on the periphery. Fiber cells are hexagonal in cross section with newly formed cells on the left (next to the epithelial cells) and maturing cells moving toward the right in concentric layers. (**C**) Scanning electron microscopy (SEM) at various depths in 3-month-old wild-type (WT) lenses (modified from [50]). Colored boxed regions indicate the approximate location where higher magnification images (lower panel) were obtained. Single fiber cells are highlighted in green as a comparison of the change in cell morphology during lens fiber maturation. Newly formed fiber cells in the cortex are straight with ball-and-socket protrusions along the broad sides and small protrusions along the short sides (red box). During maturation, differentiating fiber cells form larger protrusions along the short sides (green box). Mature inner fiber cells have large paddle domains decorated by small protrusions (blue box). These interlocking membrane interdigitations are thought to be important for mechanical stability in the lens. Scale bars, 50µm and 6µm in (**B)**.

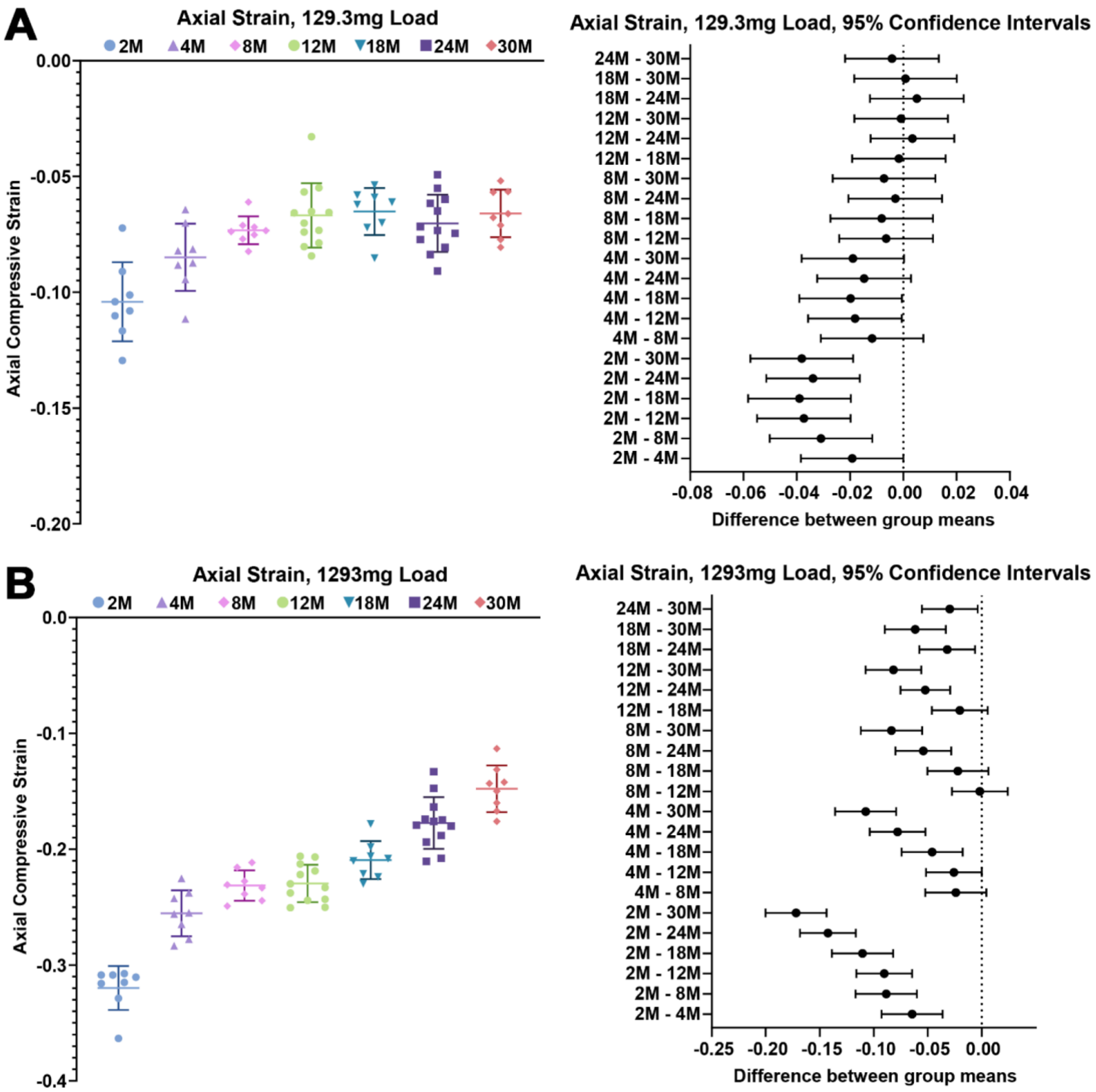

Supplementary Figure 2. Dot plots of axial strain at the lowest load (129.3mg, 1 coverslip) and the maximum load (1293mg, 10 coverslips). Lines on the plots reflect mean ± SD of n = at least 8 lenses per age. The graph below the data plots shows the 95% confidence interval. Any comparisons not crossing the dotted line are statistically significant ($p < 0.05$). (**A**, **B**) Axial strain at the lowest load (129.3mg) decreased between 2 and 8 months of age but remained unchanged after 4 months of age. Axial strain was decreased with age at the highest load (1293mg).

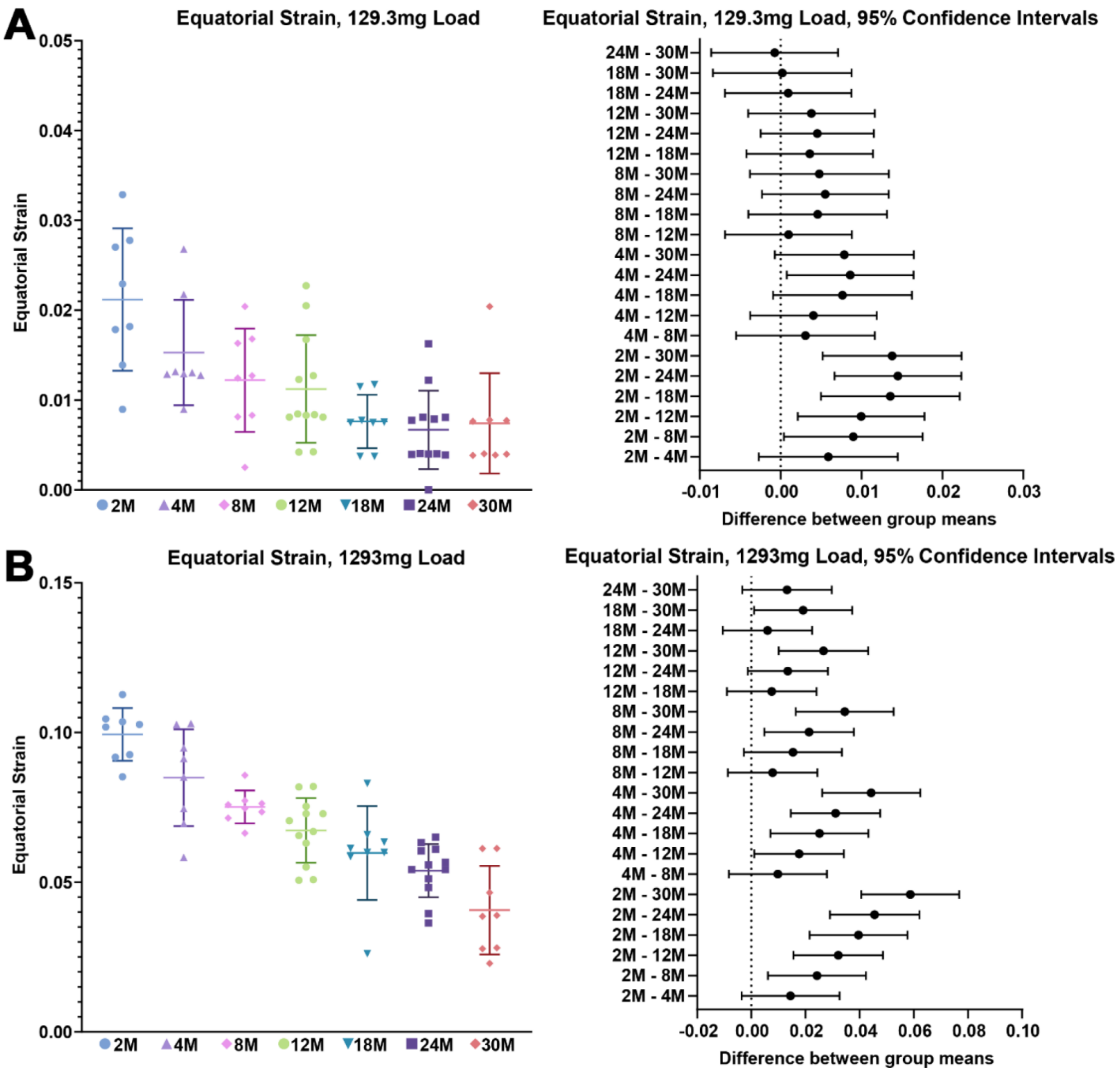

Supplementary Figure 3. Dot plots of equatorial strain at the lowest load (129.3mg, 1 coverslip) and the maximum load (1293mg, 10 coverslips). Lines on the plots reflect mean ± SD of n = at least 8 lenses per age. The graph below the data plots shows the 95% confidence interval. Any comparisons not crossing the dotted line are statistically significant ($p < 0.05$). (**A**, **B**) Equatorial strain at the lowest load was decreased between 2 and 8 months of age with no significant change after 4 months of age. Equatorial strain at the highest load was generally decreased with age. These data suggest that the lens cortex, which is compressed at the lowest load, does not stiffen much with age after 4 months.

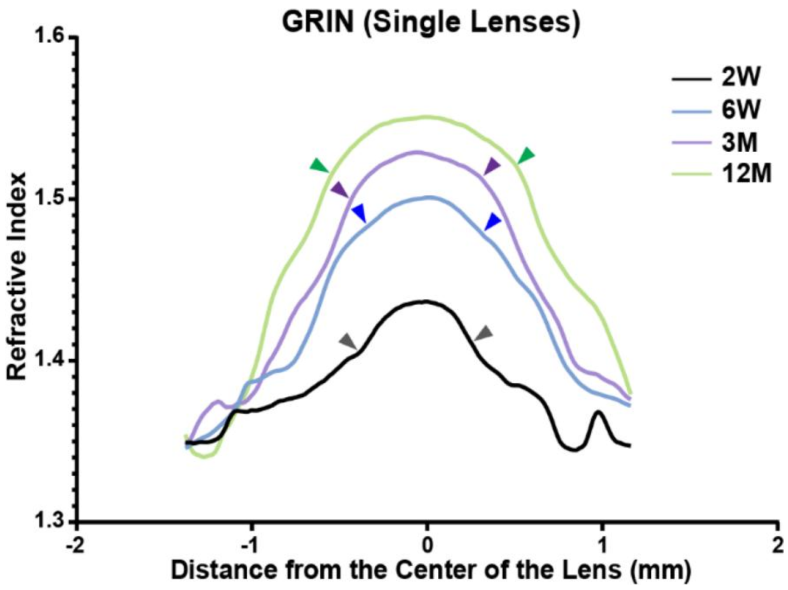

Supplementary Figure 4. Single GRIN profile plots from selected ages reveal the discontinuity in GRIN profile distinguishing the cap region from the bottom region. The curvature of the GRIN profile is therefore different in the center and periphery of the lens. The discontinuities are marked by pairs of arrowheads. The arrowheads mark the approximate location of the boundary between the nucleus and the cortex.
